# The complete chloroplast genome of Lepidium olgae, a narrow endemic species of the Nuratau Range, Uzbekistan

**DOI:** 10.12688/f1000research.179708.1

**Published:** 2026-06-01

**Authors:** Ibrokhimjon Ergashov, Farkhodjon Mingboev, Farruhbek Rasulov, Azizbek Togaev, Kibriyo Rasulova, Abdumo'min Sindorov, Muhayo Tagayeva, G‘iyos Buxorov, Ziyoviddin Yusupov, Sherzod Yakhshiboyev

**Affiliations:** 1 Institute of Botany, Academy of Sciences of Uzbekistan, Tashkent, Uzbekistan; 2 Department of Pharmaceutical Sciences, Andijan State Medical Institute, Andijan, Uzbekistan; 3 Termiz University of Economics and Service, 38-B, Ibn Sino str., Termiz region, Surkhandarya, Uzbekistan; 4 Department of Otorhinolaryngology No. 2, Samarkand State Medical University, Samarkand, Uzbekistan; 5 Jizzakh state pedagogical university, 130100, 4 Sharof Rashidov street, Jizzakh, Uzbekistan; 6 Muhammad Ikbal Street, 11, 200117, Bukhara State University, Bukhara, Uzbekistan; 7 Turon University, 1/14 Nasaf Street, Karshi City, Kashkadarya, Uzbekistan; 8 Samarkand State Architecture and Construction University, Samarkand, Uzbekistan

**Keywords:** Lepidium, chloroplast genome, phylogenetics, genetic diversity, ycf1 gene, plastome analysis, Brassicaceae

## Abstract

*Lepidium olgae* Regel is a poorly studied Central Asian species of Brassicaceae occurring in arid mountain habitats of the Nuratau Range, Uzbekistan, and its genomic resources have remained limited. In this study, we sequenced, assembled, and characterized the complete chloroplast genome of *L. olgae* and evaluated its phylogenetic position within *Lepidium* using comparative plastome data from 17 species. The plastome of *L. olgae* exhibited the typical circular quadripartite structure of angiosperms, with a total length of 154,837 base pairs. Comparative analysis showed that chloroplast genome sizes among the sampled *Lepidium* species ranged from 153,132 to 154,982 base pairs, indicating a high level of structural conservation across the genus. All analyzed plastomes contained 127 unique genes, including 82 protein-coding genes, 37 transfer ribonucleic acid genes, and 8 ribosomal ribonucleic acid genes. Gene content, gene order, and overall genome organization were highly conserved, with only minor variation detected at the boundaries of the large single-copy, small single-copy, and inverted repeat regions. Sliding-window analysis of nucleotide diversity revealed uneven sequence variation across the plastomes, with several highly variable regions, including *trnQ–psbK*, *trnD*, *trnL–trnF*, *psbJ–psbL*, *rpl14–rpl16*, *rpl32–ccsA*, and especially the *ycf1–trnN* interval. Phylogenetic analysis based on complete chloroplast genome sequences strongly supported the monophyly of *Lepidium* and recovered *L. olgae* as a distinct lineage within the genus. These results provide a useful genomic resource for *Lepidium* and establish a foundation for future phylogenetic, taxonomic, molecular identification, and conservation-related studies of Central Asian representatives of the genus.

## Introduction

The genus *Lepidium* L. (Brassicaceae), commonly known as pepperwort or peppercress, is a cosmopolitan taxon comprising between 175 and more than 260 species, depending on the circumscription and recent taxonomic revisions ( Al-Shehbaz, 2012; De Lange et al., 2013; Koch and Mummenhoff, 2006; Bona, 2020). It is widely distributed across temperate and subtropical regions globally, with centers of diversity in Central Asia, Australia, New Zealand, and the Americas ( German, 2014; Ilyinska, 2014; Rūrāne and Roze, 2019 ). Species within *Lepidium* inhabit diverse ecological niches, ranging from coastal zones to alpine environments, and display significant morphological diversity and adaptive specialization.

Historically, *Lepidium* has posed considerable challenges to systematists due to extensive morphological variation, frequent polyploidy, hybridization events, and the occurrence of both sexual and autogamous reproduction ( Al-Shehbaz, 2012; De Lange et al., 2013; Dierschke et al., 2009). The genus is characterized by small, usually inconspicuous flowers often lacking petals, and fruits that are angustiseptate silicles with a single mucilaginous seed per locule, a feature aiding in long-distance dispersal ( Koch and Mummenhoff, 2006). This combination of reproductive plasticity and dispersal capability has contributed to the broad distribution and complex taxonomy of the genus, with many species historically being grouped under broad or narrow concepts depending on regional traditions ( German, 2014).

Recent molecular phylogenetic studies have provided significant insights into the intrageneric relationships within *Lepidium*, using both nuclear markers, such as ITS and ETS, and chloroplast markers, such as *trnL-F *, *rbcL*, and *matK.* Mummenhoff et al. (2001) established the first comprehensive chloroplast DNA phylogeny for the genus, revealing a major division into three lineages largely reflecting geographic distributions: the Eurasian lineage, including sections *Lepia* and *Cardaria*; the Australasian lineage, corresponding to *Monoplocoidea*; and a widespread clade comprising the remaining taxa. These findings were supported by more recent analyses using super-barcodes, including full plastome sequences, which demonstrated that complete chloroplast genomes can provide higher phylogenetic resolution in complex genera such as *Lepidium* ( Zhou et al., 2023; Song et al., 2023).

Chloroplast genome, or plastome, sequencing has become a powerful tool for resolving phylogenetic relationships and evolutionary patterns within plant genera ( Munavvarov et al., 2022). Complete plastome sequences offer robust phylogenetic signals due to their uniparental inheritance, low recombination rates, and conserved structure; however, they also contain evolutionary hotspots that may vary among lineages ( Ergashov et al., 2026a; Ergashov et al., 2025a; Ergashov et al., 2025b; Ergashov et al., 2026b; Ergashov et al. 2026c; Jansen and Ruhlman, 2012). Plastome studies have been particularly useful in groups with intricate evolutionary histories, including cases of allopolyploidy and cryptic speciation, as observed in *Lepidium* ( Dierschke et al., 2009; Mummenhoff et al., 2004).

Despite the increasing availability of *Lepidium* plastome data in public repositories such as NCBI, comparative analyses across a broad sampling of the genus remain limited. To address this gap, the present study undertakes a comprehensive plastome-wide comparative genomic analysis across 17 *Lepidium* species. This includes one newly sequenced species, *Lepidium olgae*, endemic to arid regions of Central Asia, particularly the Nuratau region of Uzbekistan, together with 16 publicly available plastome sequences. *Lepidium olgae*, a rarely studied species with ecological adaptations to xeric environments, provides a valuable addition to the genomic sampling of the genus, particularly for understanding plastome variation in Central Asian taxa ( German, 2014; Song et al., 2023). The main objectives of this study are: (1) to characterize and compare the plastome structures, gene content, and sequence divergence among 17 *Lepidium* species; (2) to identify structural variations such as inversions, expansions and contractions of inverted repeats, and divergent hotspots; and (3) to infer phylogenetic relationships within the genus based on complete plastome data and evaluate their evolutionary implications for *Lepidium* taxonomy.

Through this comparative plastomic framework, we aim to contribute to a better understanding of chloroplast genome evolution in *Lepidium*, clarify phylogenetic relationships, and provide foundational data for future evolutionary and taxonomic studies in Brassicaceae.

## Methods

Fresh leaves of Lepidium olgae Regel were collected from a wild population in the Nuratau Range, Khayotsoy, Uzbekistan, in May 2024 (40.508056° N, 66.723889° E; Figure 1). Species identification was carried out by N. Beshko at the National Herbarium of Uzbekistan, and representative voucher specimens were deposited in the National Herbarium of Uzbekistan (TASH) under voucher accession number TASH0007.

**Figure 1.**
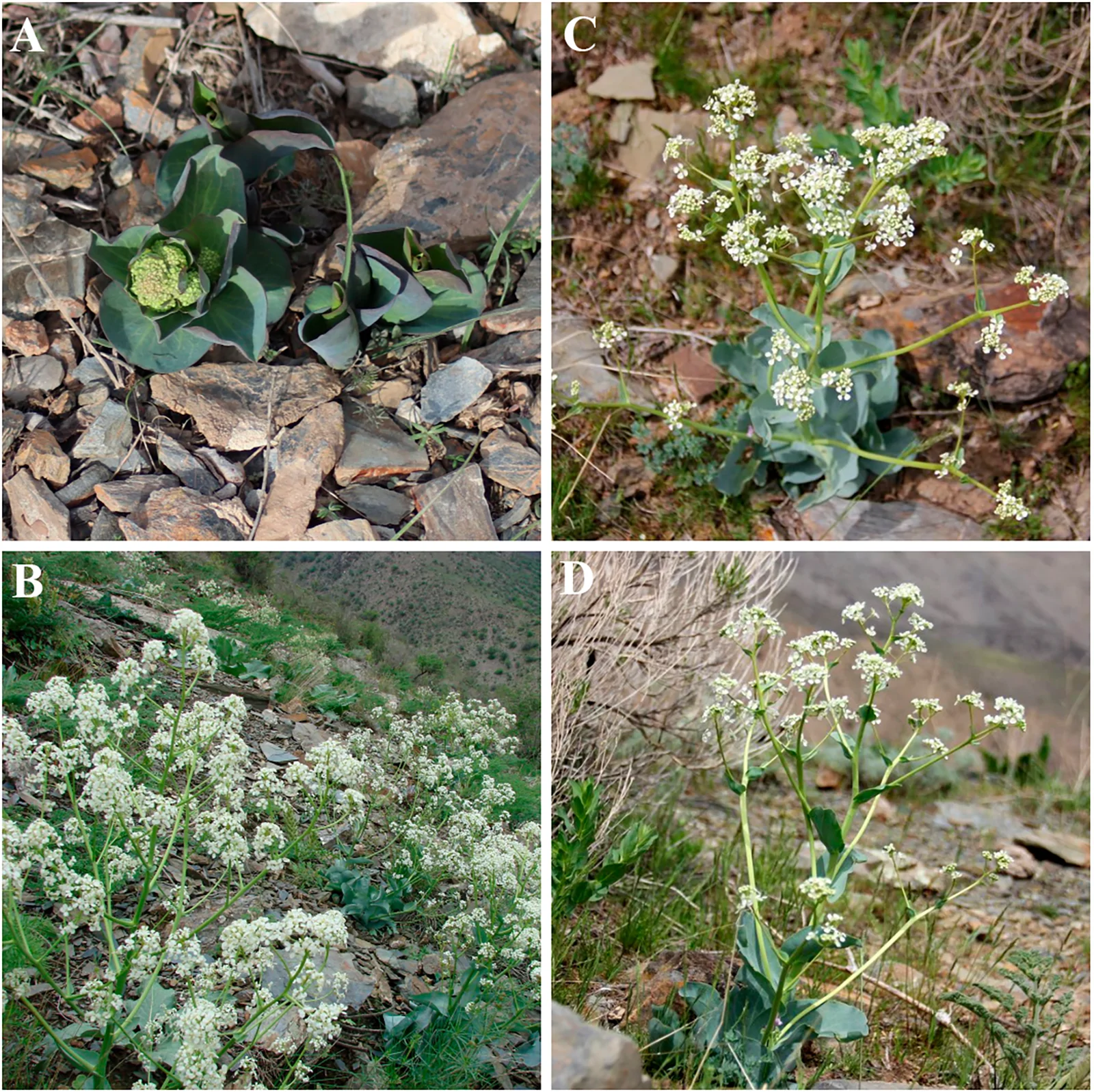
Various individuals of *L. olgae* on rocky-gravelly slopes in the Khayatsai tract, Nuratau Nature Reserve, Nuratau Range, Uzbekistan: A, plant at the beginning of the growing season, top view, approximately 1300 m a.s.l., 3 April 2022; B, flowering individual, approximately 1300 m a.s.l., 6 May 2012; C–D, flowering plants, 14 April 2013; photographs by N. Beshko.

The collected leaf material was immediately dried in silica gel and stored at room temperature until DNA extraction. Total genomic DNA was extracted from dried leaf tissue using a Tiangen plant genomic DNA extraction kit following the manufacturer’s protocol. Sequencing libraries were prepared using a NEB library preparation kit for Illumina sequencing. The genomic DNA was fragmented to approximately 350 bp, followed by end repair, adapter ligation, and PCR amplification. Library quality and fragment size distribution were assessed using an Agilent 5400 system, and library concentration was subsequently determined. Qualified libraries were sequenced on an Illumina platform at Novogene Bioinformatics Technology Co.

Clean reads were assembled using NOVOPlasty ( Dierckxsens et al., 2017). Gene annotation was performed in Geneious Prime v2025.1.2 using the chloroplast genome of *Lepidium apetalum* Willd. (GenBank accession NC_051540) as the reference. The annotation was manually checked and corrected, including verification of start and stop codons and exon–intron boundaries of protein-coding genes ( Kearse et al., 2012). The circular chloroplast genome map was generated using OGDRAW ( Greiner et al., 2019; Figure 2).

**Figure 2.**
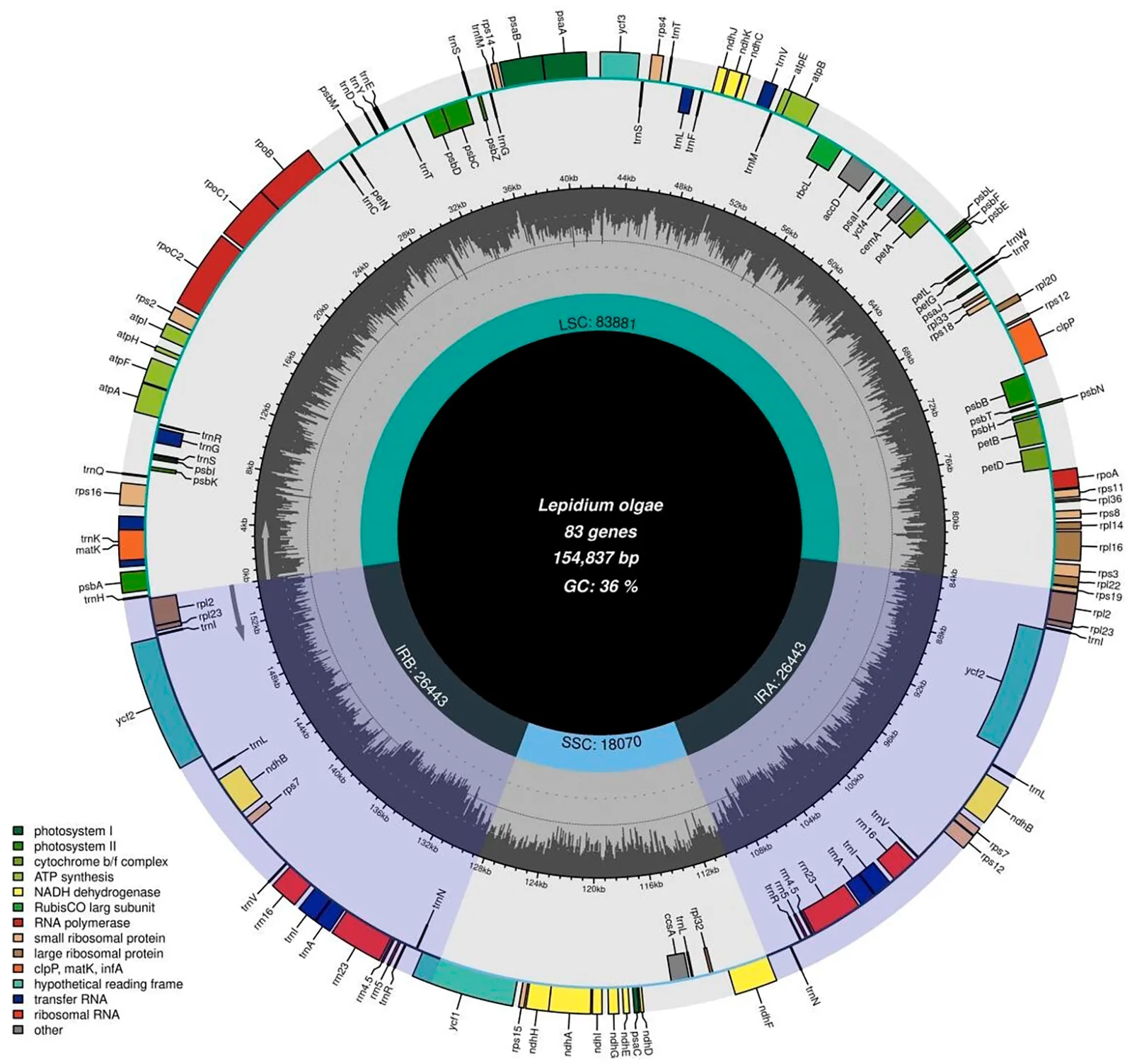
Circular map of the chloroplast genome of *Lepidium olgae.* Genes located on the outer and inner circles are transcribed in opposite directions, respectively. The inner histogram represents the GC content across the genome. IRa and IRb indicate the inverted repeat regions, while LSC and SSC denote the large single-copy and small single-copy regions, respectively.

For comparative plastome analysis, 34 chloroplast genome accessions representing 17 *Lepidium* species were retrieved from NCBI GenBank ( Table 1). Complete chloroplast genome sequences were aligned using MAFFT v7.471/v7.520 ( Katoh & Standley, 2013). Gene functions were classified into photosynthesis-related, self-replication-related, biosynthesis-related, and unknown-function categories.

**Table 1.** NCBI accession numbers of the species of the genus *Lepidium* and outgroups.

No.	Species name	NCBI accession number
1	*Hornungia petraea*	NC_049650
2	*Yinshania furcatopilosa*	MK637818
3	*Yinshania henryi*	MK637819
4	*Yinshania zayuensis*	NC_062044
5	*Lepidium echinatum*	NC_049660
6	*Lepidium perfoliatum*	OQ644480
7	*Lepidium perfoliatum*	MT880913
8	*Lepidium perfoliatum*	ON598357
9	*Lepidium draba*	NC_077506
10	*Lepidium appelianum*	NC_077510
11	*Lepidium chalepense*	NC_077508
12	*Lepidium chalepense*	ON598368
13	*Lepidium olgae*	PV605702
14	*Lepidium latifolium*	ON598362
15	*Lepidium latifolium*	ON598361
16	*Lepidium latifolium*	ON598359
17	*Lepidium* sp. XJ-151	OQ644481
18	*Lepidium cartilagineum*	NC_077509
19	*Lepidium didymum*	OY986990
20	*Lepidium meyenii*	NC_034363
21	*Lepidium meyenii*	MT430983
22	*Lepidium meyenii*	KY231152
23	*Lepidium cordatum*	NC_077507
24	*Lepidium apetalum*	NC_051540
25	*Lepidium apetalum*	OR941701
26	*Lepidium apetalum*	PP234589
27	*Lepidium ruderale*	NC_077504
28	*Lepidium ruderale*	ON598356
29	*Lepidium sativum*	NC_047178
30	*Lepidium sativum*	MK637743
31	*Lepidium virginicum*	NC_009273
32	*Lepidium ferganense*	ON598364
33	*Lepidium ferganense*	OQ644478
34	*Lepidium ferganense*	NC_077505

The structural characteristics of the chloroplast genomes, including the large single-copy region, small single-copy region, and inverted repeat regions, were compared among the sampled *Lepidium* species. Expansion and contraction of the inverted repeat regions were analyzed using IRscope ( Amiryousefi et al. 2018) and manually checked in Geneious Prime v2025.1.2. The junction regions were defined as JLB, the junction between LSC and IRb; JSB, the junction between SSC and IRb; JSA, the junction between SSC and IRa; and JLA, the junction between LSC and IRa. Variation in these boundary regions was recorded to evaluate plastome structural diversity within *Lepidium.*

Nucleotide diversity (Pi) was calculated using DnaSP v6.12.03 ( Rozas et al., 2017). A sliding-window analysis was performed with a window length of 800 bp and a step size of 200 bp to identify highly variable regions across the chloroplast genomes. Regions showing elevated Pi values were considered potential molecular markers for phylogenetic and DNA barcoding studies.

For phylogenetic analysis, 34 complete chloroplast genome sequences were used, including 30 accessions representing 16 named *Lepidium* species and one unidentified *Lepidium* accession, together with four outgroup accessions from *Hornungia* and *Yinshania* ( Table 1). Maximum likelihood analysis was performed in RAxML with 1,000 bootstrap replicates ( Stamatakis, 2014). The GTR + G model was selected using jModelTest v2.1.4 under the Akaike Information Criterion ( Darriba et al., 2012).

## Results

The chloroplast genomes of the 17 analyzed *Lepidium* species showed the typical circular quadripartite structure of angiosperm plastomes, consisting of a large single-copy (LSC) region, a small single-copy (SSC) region, and a pair of inverted repeat regions, IRa and IRb ( Figure 2). The total plastome size ranged from 153,132 bp to 154,982 bp, indicating a high level of structural conservation across the genus. The chloroplast genome of *Lepidium olgae* was 154,837 bp in length. All analyzed plastomes encoded 127 unique genes, including 82 protein-coding genes, 37 transfer RNA genes, and 8 ribosomal RNA genes. These genes were mainly associated with photosynthesis, transcription, translation, and essential metabolic processes. Gene content, gene order, and overall genomic organization were highly conserved among the examined species, and no major rearrangements were detected. This pattern agrees with previous plastome studies in Brassicaceae and other angiosperms, where conserved quadripartite structure, limited gene loss, and stable genome organization have commonly been reported ( Jansen et al., 2007; Wicke et al., 2011; Daniell et al., 2016; Huang et al., 2022; Dekhkonov et al., 2025; Tojiboeva et al., 2025; Nikitina et al., 2025).

Comparative analysis of the LSC, SSC, and IR junctions revealed only minor variation among the 17 *Lepidium* chloroplast genomes ( Figure 3). At the JLB boundary, located between LSC and IRb, the *rps19* gene was partially duplicated into the IRb region in most species, with slight differences in the length of the overlapping segment. At the JSB boundary, the *ndhF* gene consistently extended slightly into the IRb region. The JSA boundary showed limited variation, mainly related to the position and length of the *ycf1* fragment extending into IRa, while the JLA boundary was highly conserved, with adjacent genes such as *rpl2* and *psbA* maintaining stable positions. These minor expansions and contractions of IR regions are likely lineage-specific microstructural changes rather than major plastome reorganizations. Similar IR boundary shifts have been widely reported across angiosperms and are considered one of the main contributors to plastome size variation ( Zhu et al., 2016; Wang et al., 2008). The low variability of IR regions also supports the idea that duplicated regions are under stronger evolutionary constraint, probably due to copy-correction mechanisms and reduced substitution rates ( Birky and Walsh, 1992; Wicke et al., 2011; Smith, 2015).

**Figure 3.**
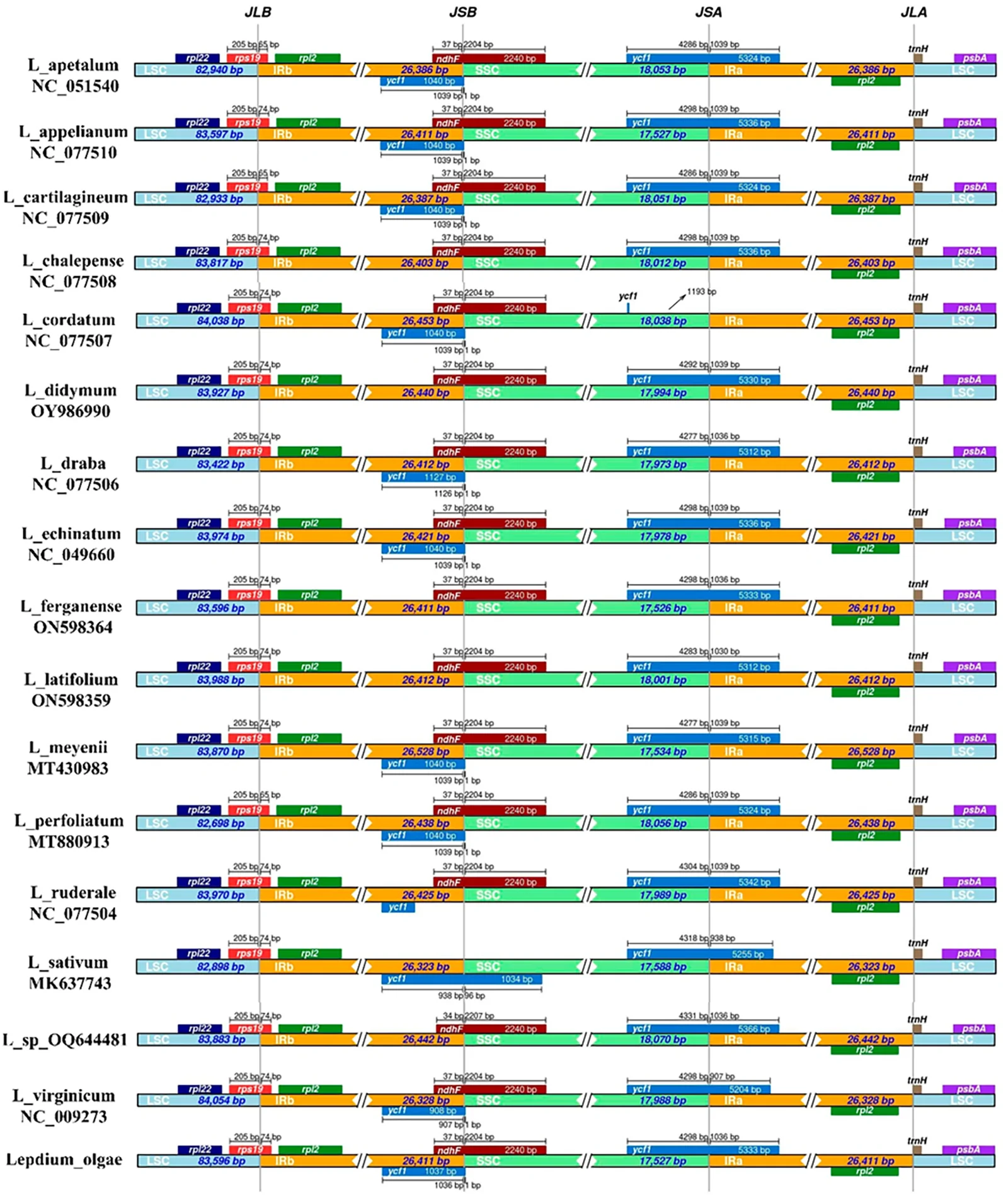
Comparative analysis of the LSC, IR, and SSC boundary regions in the chloroplast genomes of 17 *Lepidium* species. JLB represents the junction of LSC and IRb, JSB indicates the junction of SSC and IRb, JSA denotes the junction of SSC and IRa, and JLA signifies the junction of LSC and IRa.

Sliding-window analysis of nucleotide diversity showed heterogeneous sequence variation across the *Lepidium* plastomes ( Figure 4). Pi values ranged from approximately 0.002 to 0.047, indicating generally low to moderate sequence divergence among species. Several highly variable regions with Pi values above 0.03 were detected, mostly in intergenic spacers and several coding regions, including *trnQ–psbK*, *trnD*, *trnL–trnF*, *psbJ–psbL*, *rpl14–rpl16*, and *rpl32–ccsA.* The highest nucleotide diversity was observed in the *ycf1–trnN* region, where Pi values reached approximately 0.045–0.047. In contrast, IR regions showed distinctly lower nucleotide diversity than the LSC and SSC regions, reflecting the conserved nature of duplicated plastome regions.

**Figure 4.**
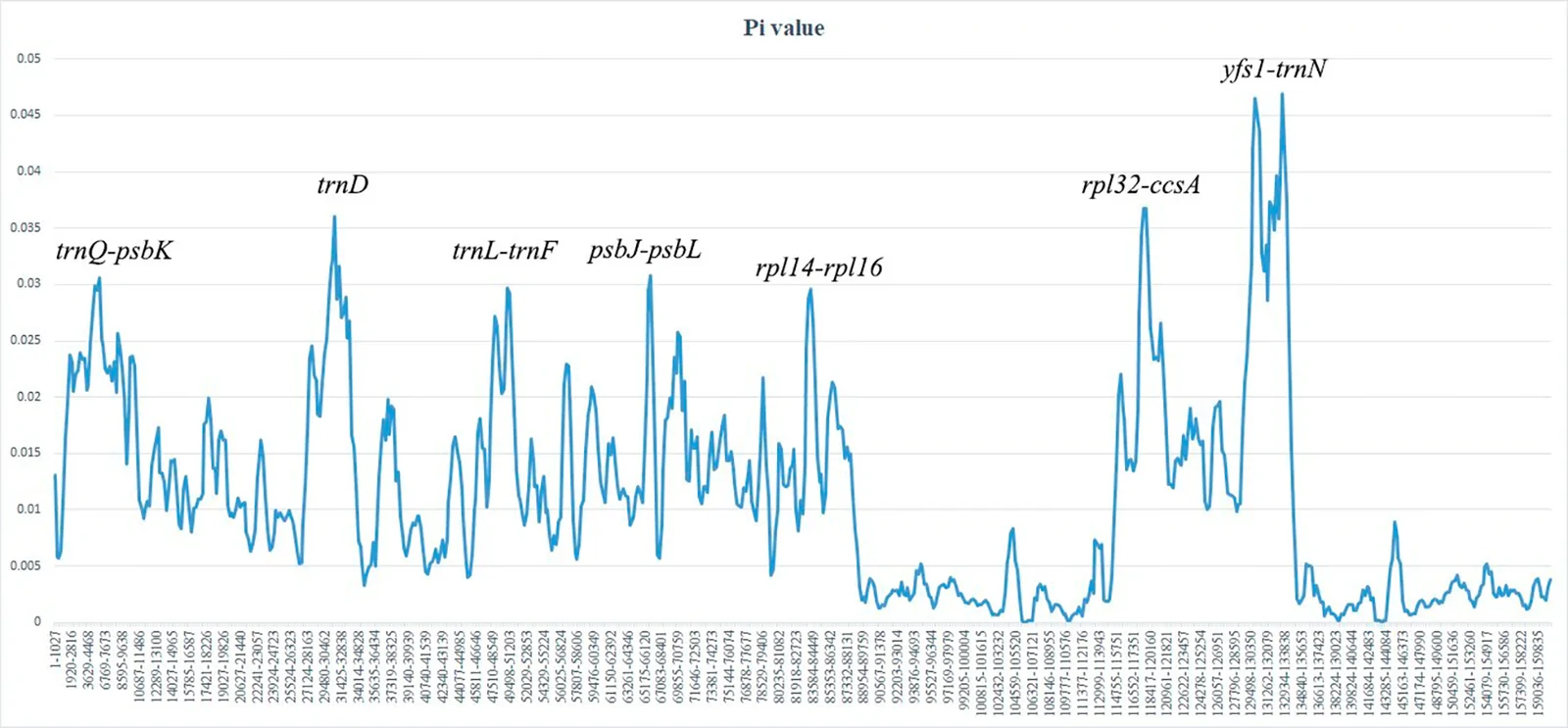
Sliding-window analysis of nucleotide diversity across *Lepidium* chloroplast genomes. Nucleotide variability (Pi) is plotted along the genome sequence. The window length was set to 800 bp with a step size of 200 bp.

The elevated variability of the *ycf1–trnN* region is consistent with previous studies showing that *ycf1* is one of the fastest-evolving regions in angiosperm plastomes and one of the most promising plastid DNA barcodes for land plants ( Jansen et al., 2007; Dong et al., 2015; Kuang et al., 2011). In addition, non-coding regions such as *rpl32–trnL-UAG *, *trnL–trnF*, and *psbJ–psbL* showed considerable variation, which is expected because intergenic spacers are generally under weaker functional constraint and accumulate substitutions more rapidly than coding regions ( Daniell et al., 2016; Shaw et al., 2007). Comparable patterns of divergence have also been reported in plastome-wide analyses of *Cardamine*, *Brassica*, and other Brassicaceae genera ( Huang et al., 2022; Ergashov et al., 2026a). Therefore, the hypervariable loci identified in the present study may serve as useful molecular markers for species delimitation, phylogenetic reconstruction, and fine-scale phylogeographic studies in *Lepidium.*

Phylogenetic analysis based on complete chloroplast genome sequences strongly supported the monophyly of *Lepidium* ( Figure 5). The maximum likelihood tree resolved major clades with bootstrap support values ranging from 67% to 100%, indicating that complete plastome data provide a robust phylogenetic signal for this genus. Multiple accessions of *L. chalepense* formed a strongly supported clade, while accessions of *L. latifolium*, *L. meyenii*, *L. apetalum*, *L. ruderale*, *L. sativum*, and *L. ferganense* clustered according to species identity. This pattern suggests high genetic consistency among accessions and confirms the usefulness of whole chloroplast genomes for resolving species-level relationships in *Lepidium.* The inclusion of related genera further supported the clear separation of *Lepidium* from closely related Brassicaceae lineages.

**Figure 5.**
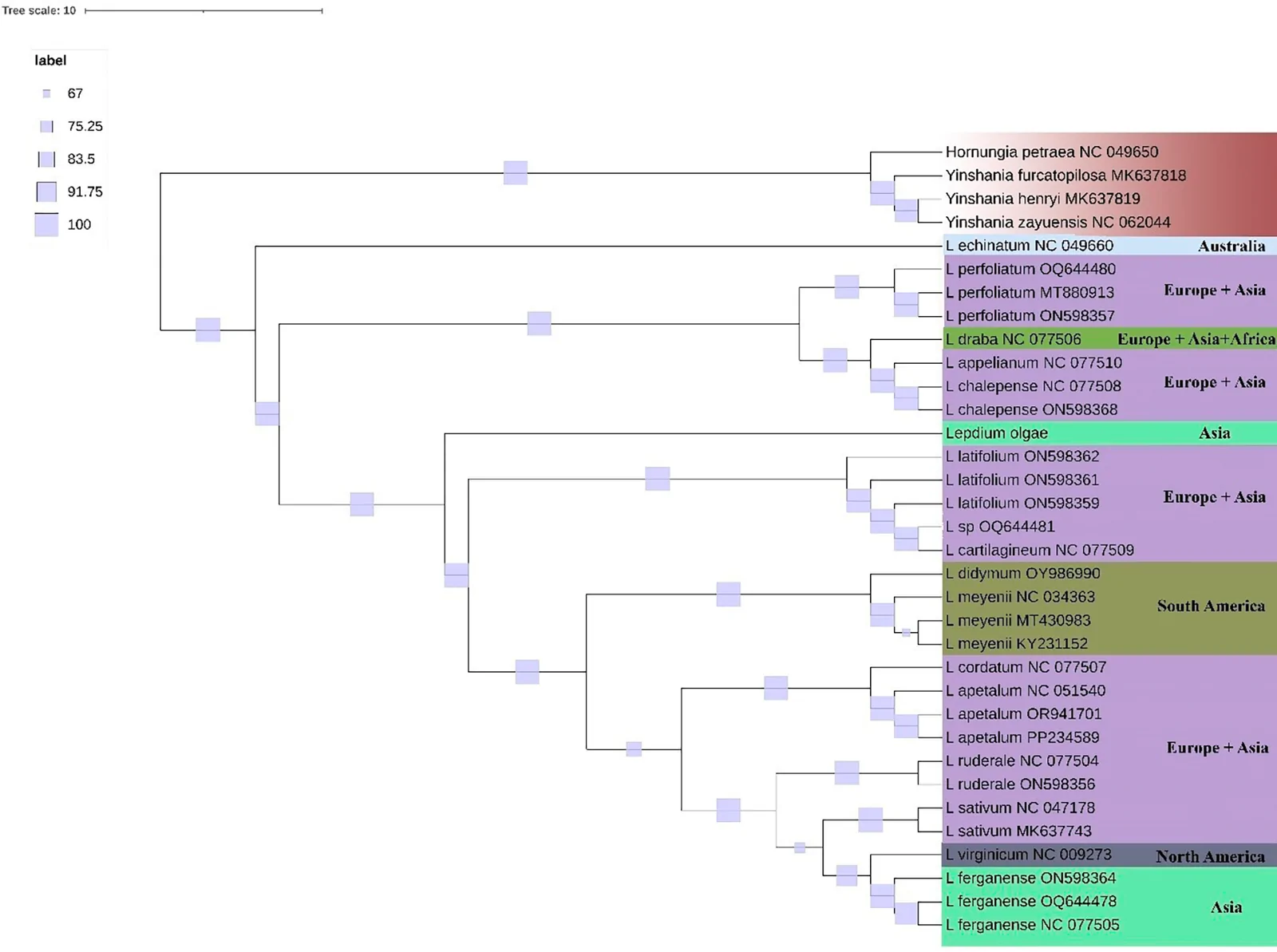
Maximum-likelihood tree of *Lepidium* plastomes. Phylogenetic relationships were inferred from complete chloroplast genome sequences. Bootstrap support values are shown at the nodes. Related genera of Brassicaceae, including *Yinshania* and *Hornungia*, were used as outgroups. .

The topology of the plastome tree broadly reflected geographic structuring within the genus. The clustering of *L. perfoliatum*, *L. draba*, *L. appelianum*, and *L. chalepense* supports the presence of a shared Eurasian lineage, in agreement with earlier chloroplast DNA phylogenies ( Mummenhoff et al., 2004; Mummenhoff et al., 2001). Similarly, South American taxa such as *L. meyenii* and *L. didymum* formed a distinct clade, suggesting independent diversification after long-distance dispersal events. The distinct placement of *L. echinatum* from Australia and the Central Asian endemic *L. olgae* highlights the importance of geographic isolation in lineage divergence. These results are consistent with biogeographic hypotheses suggesting multiple intercontinental dispersal events in *Lepidium* during the Neogene ( German, 2014; Mummenhoff et al., 2001).

The phylogenomic pattern observed here suggests that diversification in *Lepidium* has occurred mainly through geographic radiation and ecological differentiation rather than large-scale plastome restructuring. Mucilaginous seed coats, which are characteristic of *Lepidium*, may have promoted long-distance dispersal through epizoochory and water-mediated transport, facilitating colonization of new habitats ( Rūrāne and Roze, 2019; Mummenhoff et al., 2001 ). Together with predominantly autogamous reproductive systems, this dispersal strategy may have supported rapid establishment in newly colonized areas while maintaining species-level genetic cohesion. Autogamy is also associated with reduced recombination and lower within-population genetic diversity, which may partly explain the moderate plastome divergence observed in the genus ( Glémin et al., 2006; Wright et al., 2008).

Overall, the present results demonstrate that chloroplast genomes of *Lepidium* are structurally conservative, with only minor IR boundary shifts and moderate sequence divergence. At the same time, several highly variable regions, particularly *ycf1–trnN*, provide useful candidates for molecular marker development. The well-supported phylogenetic topology confirms the value of whole plastome data for clarifying taxonomic boundaries and evolutionary relationships within *Lepidium.* However, because the chloroplast genome represents a single maternally inherited locus, future studies incorporating nuclear genomic data will be necessary to resolve possible hybridization, reticulate evolution, and polyploidization events in the genus ( Birky, 2001; Mummenhoff et al., 2004). Divergence-time estimation and comparative Ka/Ks analyses of rapidly evolving genes such as *ycf1* may further clarify whether the detected hotspots reflect relaxed purifying selection or lineage-specific adaptive evolution ( Yang and Nielsen, 2000).

## Software availability

No custom code was used in this study.

Software used in the analysis included NOVOPlasty, Geneious Prime, OGDRAW, DnaSP v6.12.03, MAFFT, RAxML, and jModelTest v2.1.4, as cited in the Methods section.

## Ethics and consent

This study did not involve human participants or animals. Ethical approval and informed consent were therefore not required.

## Data Availability

NCBI GenBank: *Lepidium olgae* chloroplast genome. Accession number PV605702. The accession numbers of comparative chloroplast genomes used in this study are provided in Table 1.
