# The Complete Chloroplast Genome of *Lepidium olgae*, a Narrow Endemic Species of the Nuratau Range, Uzbekistan

**DOI:** 10.12688/f1000research.179708.2

**Published:** 2026-08-29

**Authors:** Ibrokhimjon Ergashov, Farkhodjon Mingboev, Farruhbek Rasulov, Azizbek Togaev, Kibriyo Rasulova, Abdumo'min Sindorov, Muhayo Tagayeva, G‘iyos Buxorov, Ziyoviddin Yusupov, Sherzod Yakhshiboyev

**Affiliations:** 1 Institute of Botany, Academy of Sciences of Uzbekistan, Tashkent, Uzbekistan; 2 Department of Pharmaceutical Sciences, Andijan State Medical Institute, Andijan, Uzbekistan; 3 Termiz University of Economics and Service, 38-B, Ibn Sino str., Termiz region, Surkhandarya, Uzbekistan; 4 Department of Otorhinolaryngology No. 2, Samarkand State Medical University, Samarkand, Uzbekistan; 5 Jizzakh state pedagogical university, 130100, 4 Sharof Rashidov street, Jizzakh, Uzbekistan; 6 Muhammad Ikbal Street, 11, 200117, Bukhara State University, Bukhara, Uzbekistan; 7 Turon University, 1/14 Nasaf Street, Karshi City, Kashkadarya, Uzbekistan; 8 Samarkand State Architecture and Construction University, Samarkand, Uzbekistan

**Keywords:** Lepidium, chloroplast genome, phylogenetics, genetic diversity, ycf1 gene, plastome analysis, Brassicaceae

## Abstract

*Lepidium olgae* Regel is a poorly studied narrow endemic of the Nuratau Range, Uzbekistan, for which genomic resources remain limited. In this study, we sequenced, assembled, and characterized its complete chloroplast genome and evaluated its phylogenetic position using 17 Lepidium plastome accessions representing 17 taxa, together with four outgroup plastomes. Four plastomes from *Hornungia* and *Yinshania* were included as outgroups in the phylogenetic analysis. The circular plastome of *L. olgae* was 154,837 bp in length and displayed the typical quadripartite structure, comprising an 83,881-bp large single-copy region, an 18,070-bp small single-copy region, and two inverted-repeat regions of 26,443 bp each. The overall GC content was 36.3%. Comparative analysis showed that plastome size, gene order, and overall genome organization were highly conserved among the sampled *Lepidium* taxa, with only limited variation at the single-copy and inverted-repeat junctions. Feature-level nucleotide-diversity analysis identified *trnG-GCC–trnR-UCU* and *rpl32–trnL-UAG* among the most variable non-coding regions, whereas *ycf1* showed the highest diversity among the examined coding regions. Maximum-likelihood analysis of 21 complete plastomes placed *L.* olgae as a distinct lineage within the sampled Asian *Lepidium* assemblage. The newly generated plastome provides an important genomic reference for *L. olgae*, while the identified variable regions represent candidate loci for future taxonomic, phylogeographic, and population-level studies. Broader taxonomic sampling and nuclear genomic data will be required to clarify the evolutionary history of the genus and assess the conservation genetics of this narrow endemic species.

## Introduction

The genus *Lepidium* L. (Brassicaceae), commonly known as pepperwort or peppercress, is a cosmopolitan taxon comprising between 175 and more than 260 species, depending on the circumscription and recent taxonomic revisions ( Al-Shehbaz, 2012; De Lange et al., 2013; Koch and Mummenhoff, 2006; Bona, 2020). It is widely distributed across temperate and subtropical regions globally, with centers of diversity in Central Asia, Australia, New Zealand, and the Americas ( German, 2014; Ilyinska, 2014; Rūrāne and Roze, 2019 ). Species within *Lepidium* inhabit diverse ecological niches, ranging from coastal zones to alpine environments, and display significant morphological diversity and adaptive specialization.

Historically, *Lepidium* has posed considerable challenges to systematists due to extensive morphological variation, frequent polyploidy, hybridization events, and the occurrence of both sexual and autogamous reproduction ( Al-Shehbaz, 2012; De Lange et al., 2013; Dierschke et al., 2009). The genus is characterized by small, usually inconspicuous flowers often lacking petals, and fruits that are angustiseptate silicles with a single mucilaginous seed per locule, a feature aiding in long-distance dispersal ( Koch and Mummenhoff, 2006). This combination of reproductive plasticity and dispersal capability has contributed to the broad distribution and complex taxonomy of the genus, with many species historically being grouped under broad or narrow concepts depending on regional traditions ( German, 2014).

Recent molecular phylogenetic studies have provided significant insights into the intrageneric relationships within *Lepidium*, using both nuclear markers, such as ITS and ETS, and chloroplast markers, such as *trnL-F *, *rbcL*, and *matK.* Mummenhoff et al. (2001) established the first comprehensive chloroplast DNA phylogeny for the genus, revealing a major division into three lineages largely reflecting geographic distributions: the Eurasian lineage, including sections *Lepia* and *Cardaria*; the Australasian lineage, corresponding to *Monoplocoidea*; and a widespread clade comprising the remaining taxa. These findings were supported by more recent analyses using super-barcodes, including full plastome sequences, which demonstrated that complete chloroplast genomes (cp genomes) can provide higher phylogenetic resolution in complex genera such as *Lepidium* ( Zhou et al., 2023; Song et al., 2023).

Cp genome, or plastome, sequencing has become a powerful tool for resolving phylogenetic relationships and evolutionary patterns within plant genera ( Munavvarov et al., 2022). Complete plastome sequences offer robust phylogenetic signals due to their uniparental inheritance, low recombination rates, and conserved structure; however, they also contain evolutionary hotspots that may vary among lineages ( Ergashov et al., 2025; Jansen and Ruhlman, 2012). Plastome studies have been particularly useful in groups with intricate evolutionary histories, including cases of allopolyploidy and cryptic speciation, as observed in *Lepidium* ( Dierschke et al., 2009; Mummenhoff et al., 2004).

Despite the increasing availability of *Lepidium* plastome data in public repositories such as NCBI, comparative analyses across a broad sampling of the genus remain limited. To address this gap, the present study undertakes a comprehensive plastome-wide comparative genomic analysis across 17 *Lepidium* species. This includes one newly sequenced species, *Lepidium olgae*, endemic to arid regions of Central Asia, particularly the Nuratau region of Uzbekistan, together with 16 publicly available plastome sequences. *Lepidium olgae*, a rarely studied species with ecological adaptations to xeric environments, provides a valuable addition to the genomic sampling of the genus, particularly for understanding plastome variation in Central Asian taxa ( German, 2014; Song et al., 2023). The main objectives of this study are: (1) to characterize and compare the plastome structures, gene content, and sequence divergence among 17 *Lepidium* species; (2) to identify structural variations such as inversions, expansions and contractions of inverted repeats, and divergent hotspots; and (3) to infer phylogenetic relationships within the genus based on complete plastome data and evaluate their evolutionary implications for *Lepidium* taxonomy.

Through this comparative plastomic framework, we aim to contribute to a better understanding of cp genome evolution in *Lepidium*, clarify phylogenetic relationships, and provide foundational data for future evolutionary and taxonomic studies in Brassicaceae.

## Methods

Fresh leaves of Lepidium olgae Regel were collected from a wild population in the Nuratau Range, Khayotsoy, Uzbekistan, in May 2024 (40.508056° N, 66.723889° E; Figure 1). Species identification was carried out by N. Beshko at the National Herbarium of Uzbekistan, and representative voucher specimens were deposited in the National Herbarium of Uzbekistan (TASH) under voucher accession number TASH0007.

**Figure 1.**
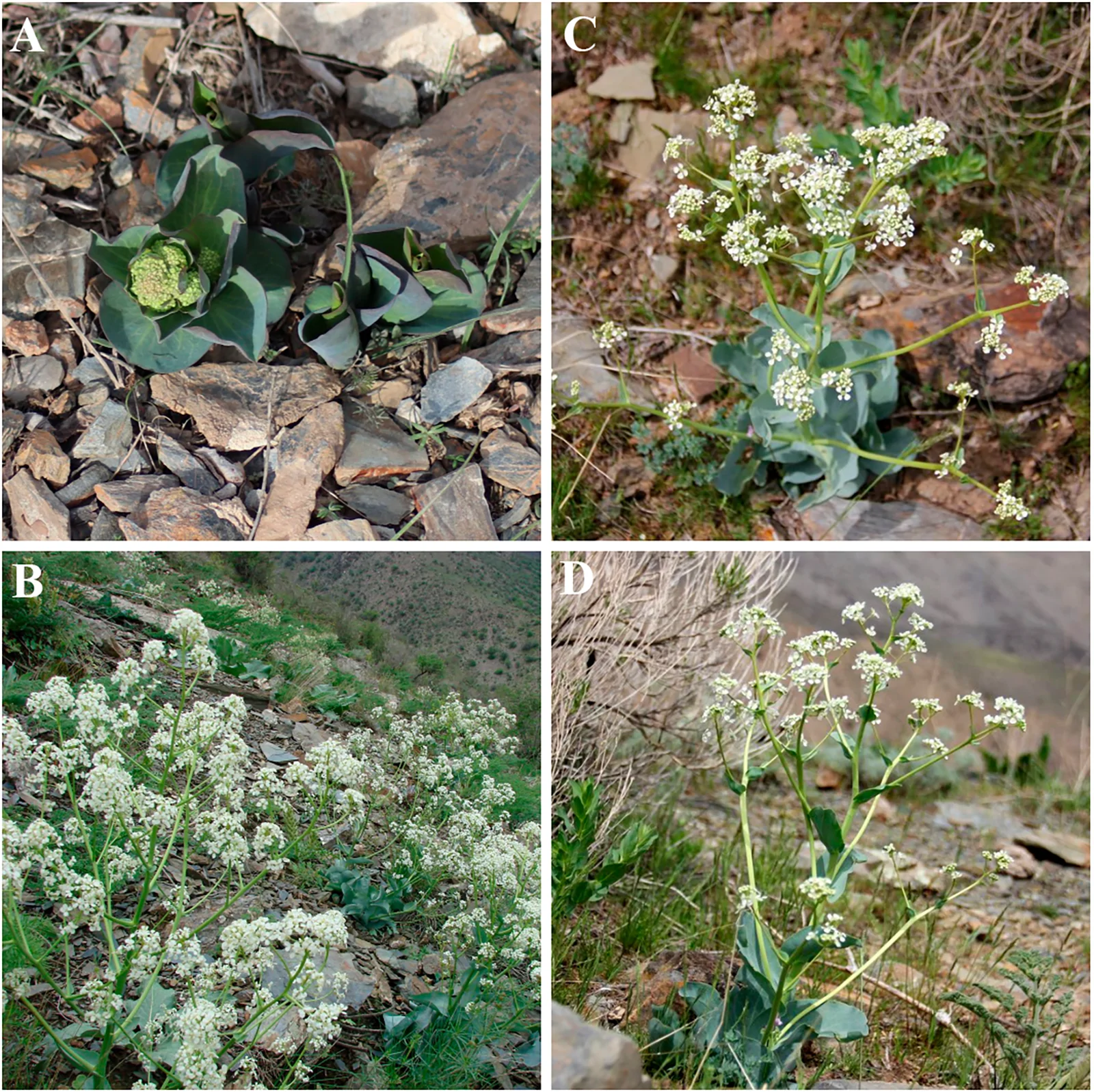
Various individuals of *L. olgae* on rocky-gravelly slopes in the Khayatsai tract, Nuratau Nature Reserve, Nuratau Range, Uzbekistan. A, plant at the beginning of the growing season, top view, approximately 1300 m a.s.l., 3 April 2022; B, flowering individual, approximately 1300 m a.s.l., 6 May 2012; C–D, flowering plants, 14 April 2013; photographs by N. Beshko.

The collected leaf material was immediately dried in silica gel and stored at room temperature until DNA extraction. Total genomic DNA was extracted from dried leaf tissue using a Tiangen plant genomic DNA extraction kit following the manufacturer’s protocol. Sequencing libraries were prepared using a NEB library preparation kit for Illumina sequencing. The genomic DNA was fragmented to approximately 350 bp, followed by end repair, adapter ligation, and PCR amplification. Library quality and fragment size distribution were assessed using an Agilent 5400 system, and library concentration was subsequently determined. Qualified libraries were sequenced by Novogene Bioinformatics Technology Co. on an Illumina NovaSeq 6000 platform using a 150-bp paired-end sequencing configuration.

The quality-filtered reads were assembled de novo using NOVOPlasty ( Dierckxsens et al., 2017), with the default k-mer value of 33. The cp genome of *Lepidium apetalum* (GenBank accession NC_051540) was used as the reference plastome for seed selection. Assembly parameters not specifically stated were maintained at their default settings.

To ensure reproducibility and public access to the underlying data, the raw paired-end sequencing reads have now been deposited in the NCBI Sequence Read Archive. The data are associated with BioProject PRJNA1499946 and BioSample SAMN61898456 and are available under SRA run accession SRR39796388. The assembled and annotated cp genome remains available in GenBank under accession PV605702.

Gene annotation was performed in Geneious Prime v2025.1.2 using the cp genome of *Lepidium apetalum* Willd. (GenBank accession NC_051540) as the reference. The annotation was manually checked and corrected, including verification of start and stop codons and exon–intron boundaries of protein-coding genes ( Kearse et al., 2012). The circular cp genome map was generated using OGDRAW ( Greiner et al., 2019; Figure 2).

**Figure 2.**
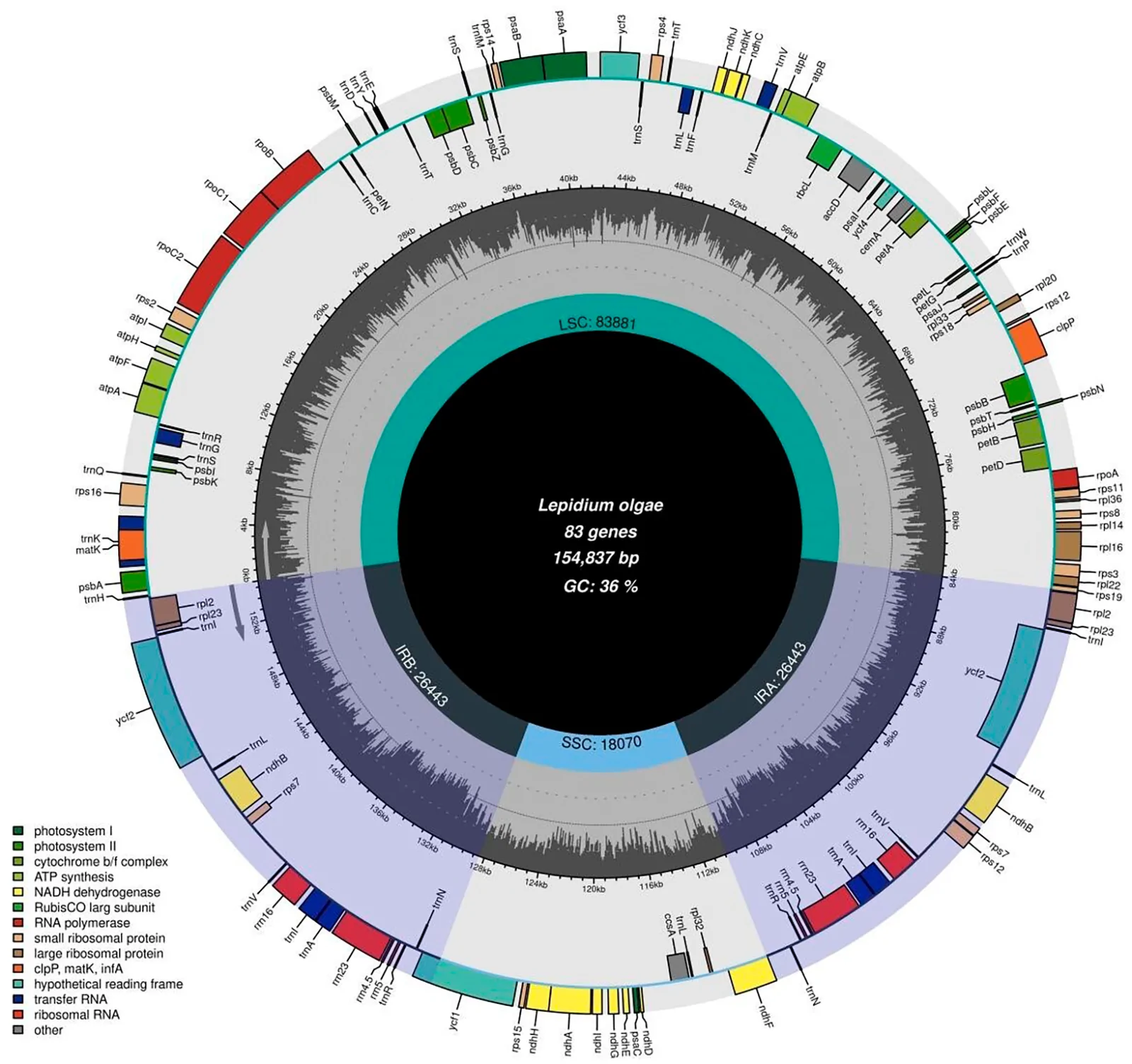
Circular map of the cp genome of *Lepidium olgae.* Genes shown outside the outer circle are transcribed counterclockwise, whereas genes shown inside the circle are transcribed clockwise. Genes are color-coded according to their functional categories, as indicated in the legend. The inner gray histogram represents variation in local GC content across the plastome, with the height of the histogram corresponding to the relative GC percentage; the lighter background represents AT content. The inner colored ring indicates the large single-copy region (LSC), small single-copy region (SSC), and the two inverted-repeat regions (IRa and IRb).

Seventeen complete plastome accessions representing 17 *Lepidium* taxa were retrieved from NCBI GenBank and included in the comparative analyses. Four additional plastomes from *Hornungia* and *Yinshania* were included as outgroups in the phylogenetic analysis, resulting in a total dataset of 21 accessions ( Table 1). The complete plastome sequences were aligned using MAFFT v7.471/v7.520 ( Katoh and Standley, 2013). Annotated genes were classified into functional categories related to photosynthesis, self-replication, biosynthesis, and genes of unknown function.

**Table 1.** Plastome accessions retained for comparative and phylogenetic analyses of *Lepidium* and the selected outgroups.

No.	Species name	NCBI accession number
1	*Hornungia petraea*	NC_049650
2	*Yinshania furcatopilosa*	MK637818
3	*Yinshania henryi*	MK637819
4	*Yinshania zayuensis*	NC_062044
5	*Lepidium echinatum*	NC_049660
6	*Lepidium perfoliatum*	OQ644480
7	*Lepidium draba*	NC_077506
8	*Lepidium appelianum*	NC_077510
9	*Lepidium chalepense*	NC_077508
10	*Lepidium olgae*	PV605702
11	*Lepidium latifolium*	ON598362
12	*Lepidium* sp. XJ-151	OQ644481
13	*Lepidium cartilagineum*	NC_077509
14	*Lepidium didymum*	OY986990
15	*Lepidium meyenii*	NC_034363
16	*Lepidium cordatum*	NC_077507
17	*Lepidium apetalum*	NC_051540
18	*Lepidium ruderale*	NC_077504
19	*Lepidium sativum*	NC_047178
20	*Lepidium virginicum*	NC_009273
21	*Lepidium ferganense*	NC_077505

The structural characteristics of the cp genomes, including the large single-copy region, small single-copy region, and inverted repeat regions, were compared among the sampled *Lepidium* species. Expansion and contraction of the inverted repeat regions were analyzed using IRscope ( Amiryousefi et al., 2018) and manually checked in Geneious Prime v2025.1.2. The junction regions were defined as JLB, the junction between LSC and IRb; JSB, the junction between SSC and IRb; JSA, the junction between SSC and IRa; and JLA, the junction between LSC and IRa. Variation in these boundary regions was recorded to evaluate plastome structural diversity within *Lepidium.*

The nucleotide diversity of CDS, tRNA, rRNA, introns, and intergenic spacer regions were determined based on the analysis of all 21 cp genomes included in this study using CGAS v 1.0.1 ( Abdullah et al., 2026).

Phylogenetic analysis was performed using 21 complete plastome sequences, including 17 *Lepidium* accessions—representing 16 named species and one unidentified accession, *Lepidium* sp. XJ-151—and four outgroup accessions from *Hornungia* and *Yinshania* ( Table 1). Maximum likelihood analysis was performed in RAxML with 1,000 bootstrap replicates ( Stamatakis, 2014). The GTR + G model was selected using jModelTest v2.1.4 under the Akaike Information Criterion ( Darriba et al., 2012).

## Results

The cp genomes of the 17 analyzed *Lepidium* species showed the typical circular quadripartite structure of angiosperm plastomes, consisting of a large single-copy (LSC) region, a small single-copy (SSC) region, and a pair of inverted repeat regions, IRa and IRb ( Figure 2). The total plastome size ranged from 153,132 bp to 154,982 bp, indicating a high level of structural conservation across the genus. The cp genome of *Lepidium olgae* was 154,837 bp in length. All analyzed plastomes encoded 128 unique genes, including 83 protein-coding genes, 37 transfer RNA genes, and 8 ribosomal RNA genes ( Tables 2 and 3). These genes were mainly associated with photosynthesis, transcription, translation, and essential metabolic processes. Gene content, gene order, and overall genomic organization were highly conserved among the examined species, and no major rearrangements were detected. This pattern agrees with previous plastome studies in Brassicaceae and other angiosperms, where conserved quadripartite structure, limited gene loss, and stable genome organization have commonly been reported ( Jansen et al., 2007; Wicke et al., 2011; Daniell et al., 2016; Huang et al., 2022; Dekhkonov et al., 2025; Tojiboeva et al., 2025; Nikitina et al., 2025).

**Table 2.** Genes annotated in the cp genome of *Lepidium olgae*, grouped according to functional category.

Category	Functional group	Genes
Self-replication	Large-subunit ribosomal proteins	*rpl2* ^b^ ^c^ (×2), *rpl14, rpl16* ^b^, *rpl20, rpl22, rpl23* ^c^ (×2), *rpl32, rpl33, rpl36*
Self-replication	Small-subunit ribosomal proteins	*rps2, rps3, rps4, rps7* ^c^ (×2), *rps8, rps11, rps12* ^c^ (×2), *rps14, rps15, rps16* ^b^, *rps18, rps19*
Self-replication	DNA-dependent RNA polymerase	*rpoA, rpoB, rpoC1* ^b^, *rpoC2*
Self-replication	Ribosomal RNA genes	*rrn4.5* ^c^ (×2), *rrn5* ^c^ (×2), *rrn16* ^c^ (×2), *rrn23* ^c^ (×2)
Self-replication	Transfer RNA genes	*trnA-UGC* ^b^ ^c^ (×2), *trnC-GCA, trnD-GUC, trnE-UUC, trnF-GAA, trnfM-CAU, trnG-UCC, trnH-GUG, trnI-CAU* ^c^ (×2), *trnI-GAU* ^b^ ^c^ (×2), *trnG-GCC, trnK-UUU* ^b^, *trnL-CAA* ^c^ (×2), *trnL-UAA* ^b^, *trnL-UAG, trnM-CAU, trnN-GUU* ^c^ (×2), *trnQ-UUG, trnP-UGG, trnR-ACG* ^c^ (×2), *trnR-UCU, trnS-GCU, trnS-GGA, trnS-UGA, trnT-GGU, trnT-UGU, trnV-GAC* ^c^ (×2), *trnV-UAC* ^b^, *trnW-CCA, trnY-GUA*
Photosynthesis	Photosystem I subunits	*psaA, psaB, psaC, psaI, psaJ*
Photosynthesis	Photosystem II subunits	*psbA, psbB, psbC, psbD, psbE, psbF, psbH, psbI, psbJ, psbK, psbL, psbM, psbN, psbT, psbZ*
Photosynthesis	NADH dehydrogenase subunits	*ndhA* ^b^, *ndhB* ^b^ ^c^ (×2), *ndhC, ndhD, ndhE, ndhF, ndhG, ndhH, ndhI, ndhJ, ndhK*
Photosynthesis	Cytochrome *b*6/ *f* complex	*petA, petB* ^b^, *petD* ^b^, *petG, petL, petN*
Photosynthesis	ATP synthase subunits	*atpA, atpB, atpE, atpF* ^b^, *atpH, atpI*
Photosynthesis	Rubisco large subunit	*rbcL*
Other functions	Group II intron maturase	*matK*
Other functions	Acetyl-CoA carboxylase subunit	*accD*
Other functions	Envelope membrane protein	*cemA*
Other functions	Protease	*clpP* ^a^
Other functions	Cytochrome *c* synthesis protein	*ccsA*

*Note:* ^a^Gene containing two introns; ^b^gene containing one intron; ^c^gene duplicated in the inverted-repeat regions; ×2 indicates two copies in the plastome.

**Table 3.** Structural and nucleotide-composition features of the *Lepidium olgae* cp genome.

Feature	Value
Total cp genome length (bp)	154,837
Large single-copy region length (bp)	83,881
Small single-copy region length (bp)	18,070
Inverted-repeat region length (bp, each copy)	26,443
Total GC content (%)	36.3
LSC GC content (%)	34.0
SSC GC content (%)	29.0
IR GC content (%)	42.3
Total number of annotated genes	130
Protein-coding genes	83
Transfer RNA genes	37
Ribosomal RNA genes	8

**Abbreviations:** LSC, large single-copy region; SSC, small single-copy region; IR, inverted-repeat region. The IR length and GC content refer to each individual IR copy.

**Figure 4.**
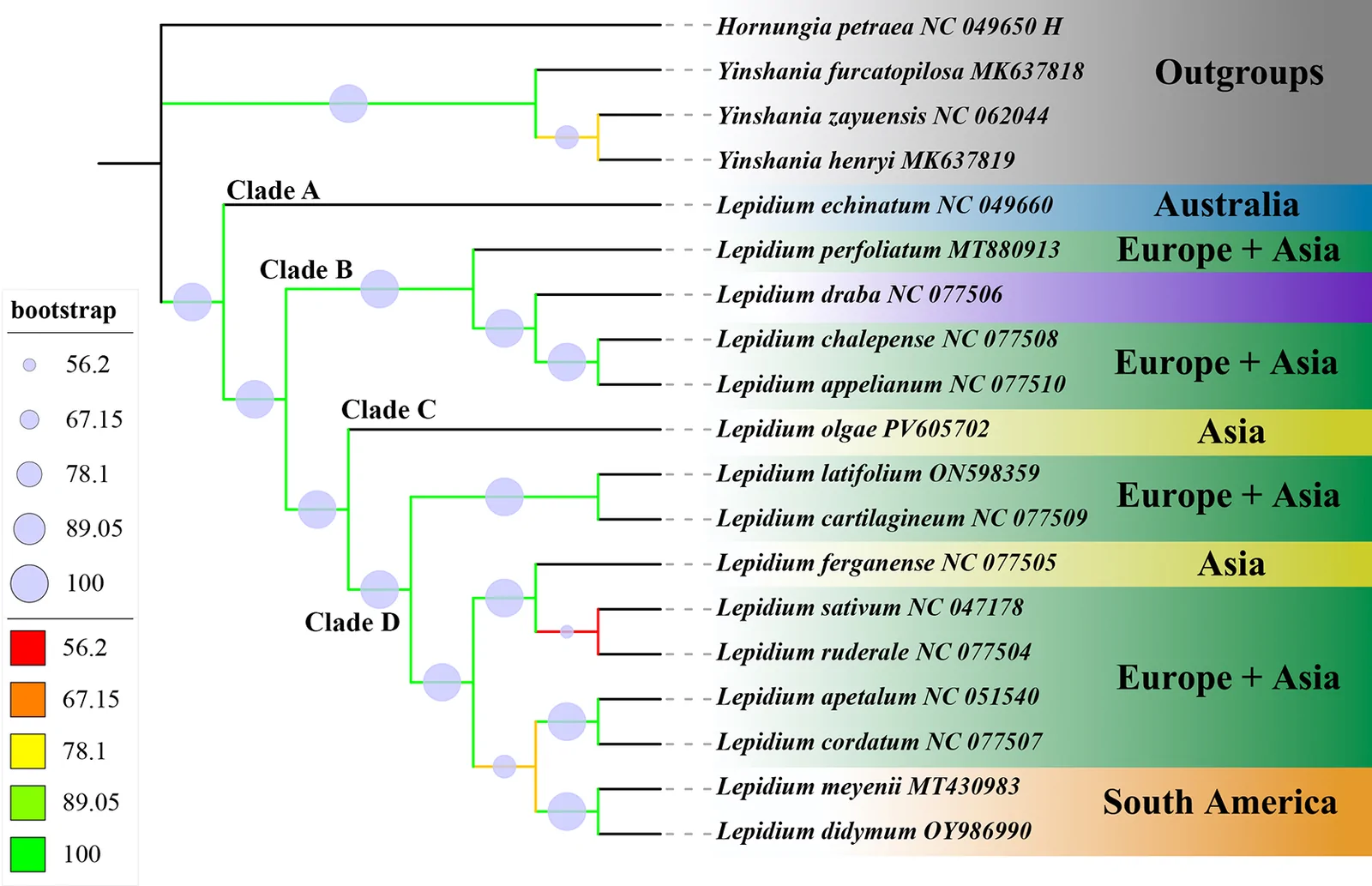
Maximum-likelihood tree of *Lepidium* plastomes. Phylogenetic relationships were inferred from complete cp genome sequences. Bootstrap support values are shown at the nodes. Related genera of Brassicaceae, including *Yinshania* and *Hornungia*, were used as outgroups.

Sliding window analysis (window length: 800 bp; step size: 200 bp) revealed heterogeneous distribution of nucleotide diversity (Pi) across the chloroplast genomes ( Figure 3). Pi values ranged from approximately 0.002 to 0.047, indicating generally low to moderate sequence divergence among species. Several highly variable regions (Pi > 0.03) were identified, primarily within intergenic spacers and certain coding regions. These included trnQ–psibKi, trnD, trnL–trnF, psbJ–psbL, rpl14–rpl16, and rpl32–ccsA. The most variable region was located in the ycf1–trnN interval, where Pi values reached up to 0.045–0.047.

**Figure 3.**
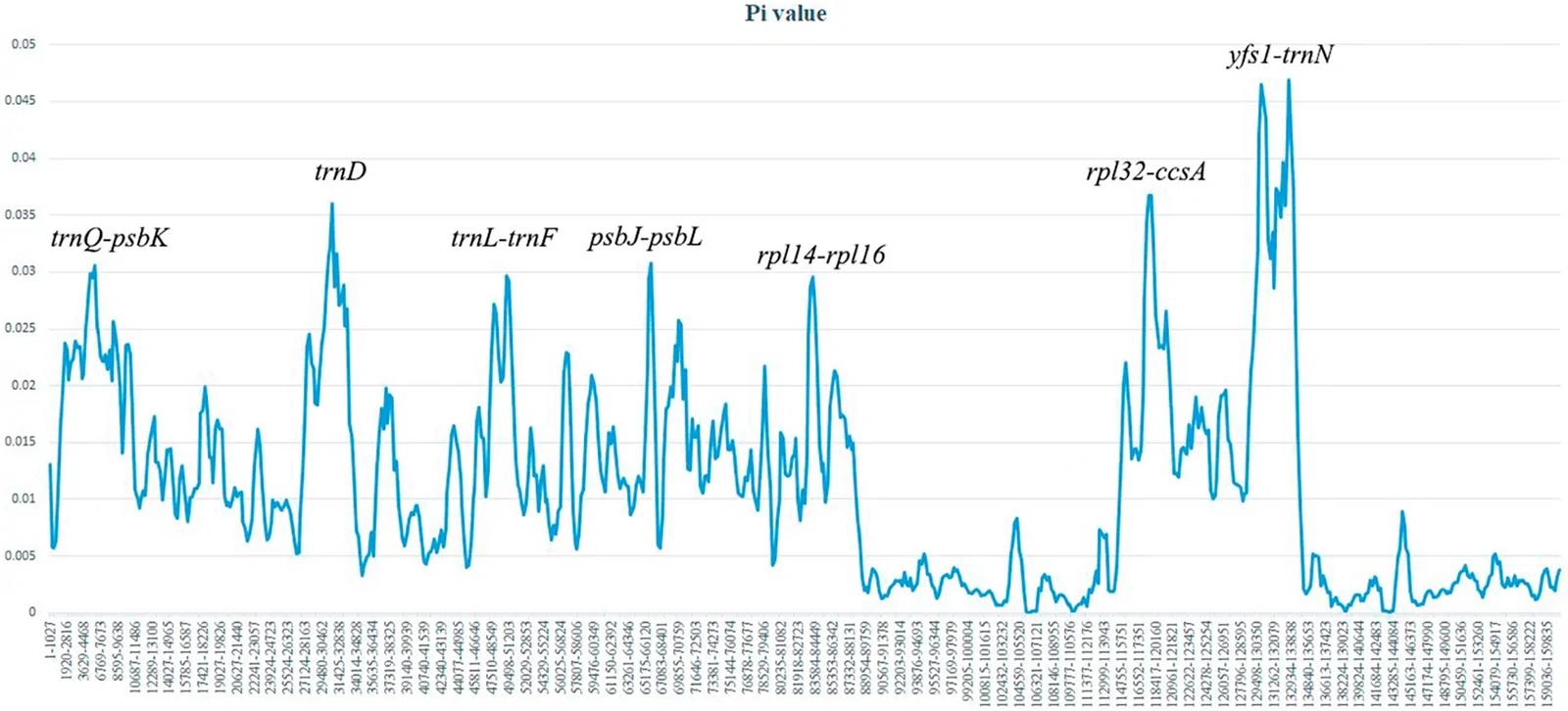
Sliding-window analysis of nucleotide diversity across *Lepidium* cp genomes.

The elevated variability of the *ycf1* coding region is consistent with previous studies showing that *ycf1* is one of the fastest-evolving regions in angiosperm plastomes and one of the most promising plastid DNA barcodes for land plants ( Jansen et al., 2007; Dong et al., 2015; Kuang et al., 2011). In addition, non-coding regions such as *trnG-GCC–trnR-UCU* and *rpl32–trnL-UAG* showed considerable variation, which is expected because intergenic spacers are generally under weaker functional constraint and accumulate substitutions more rapidly than coding regions ( Daniell et al., 2016; Shaw et al., 2007). Comparable patterns of divergence have also been reported in plastome-wide analyses of *Cardamine*, *Brassica*, and other Brassicaceae genera ( Huang et al., 2022). Therefore, the hypervariable loci identified in the present study may serve as useful molecular markers for species delimitation, phylogenetic reconstruction, and fine-scale phylogeographic studies in *Lepidium.*

Phylogenetic analysis based on complete cp genome sequences strongly supported the monophyly of *Lepidium* ( Figure 4). The maximum likelihood tree resolved major clades with bootstrap support values ranging from 67% to 100%, indicating that complete plastome data provide a robust phylogenetic signal for this genus. The phylogenetic tree resolved four main clades. Clade A contained *Lepidium echinatum*, whereas Clade B included *L. perfoliatum*, *L. draba*, *L. chalepense*, and *L. appelianum*. *Lepidium olgae* was placed in Clade C. Clade D comprised the remaining Eurasian and South American species. *Lepidium echinatum* formed the earliest-diverging lineage, while *L. olgae* occupied a distinct position within the main Asian clade. The remaining species were grouped largely according to their geographic distributions, with Eurasian and South American taxa forming separate subclades.

The topology of the plastome tree broadly reflected geographic structuring within the genus. The clustering of *L. perfoliatum*, *L. draba*, *L. appelianum*, and *L. chalepense* supports the presence of a shared Eurasian lineage, in agreement with earlier chloroplast DNA phylogenies ( Mummenhoff et al., 2001, 2004). Similarly, South American taxa such as *L. meyenii* and *L. didymum* formed a distinct clade, suggesting independent diversification after long-distance dispersal events. The distinct placement of *L. echinatum* from Australia and the Central Asian endemic *L. olgae* highlights the importance of geographic isolation in lineage divergence. These results are consistent with biogeographic hypotheses suggesting multiple intercontinental dispersal events in *Lepidium* during the Neogene ( German, 2014; Mummenhoff et al., 2001).

The phylogenomic pattern observed here is consistent with a role for geographic radiation and ecological differentiation in the diversification of *Lepidium*. Mucilaginous seed coats, which are characteristic of the genus, may facilitate dispersal through animals and water and thereby contribute to the colonization of new habitats ( Rūrāne and Roze, 2019; Mummenhoff et al., 2001 ).

Overall, the present results demonstrate that cp genomes of *Lepidium* are structurally conservative, with only minor IR boundary shifts and moderate sequence divergence. At the same time, several highly variable regions, particularly *ycf1–trnN*, provide useful candidates for molecular marker development. The well-supported phylogenetic topology confirms the value of whole plastome data for clarifying taxonomic boundaries and evolutionary relationships within *Lepidium.* However, because the cp genome represents a single maternally inherited locus, future studies incorporating nuclear genomic data will be necessary to resolve possible hybridization, reticulate evolution, and polyploidization events in the genus ( Birky, 2001; Mummenhoff et al., 2004). Divergence-time estimation and comparative Ka/Ks analyses of rapidly evolving genes such as *ycf1* may further clarify whether the detected hotspots reflect relaxed purifying selection or lineage-specific adaptive evolution ( Yang and Nielsen, 2000).

## Software availability

No custom code was used in this study.

Software used in the analysis included NOVOPlasty, Geneious Prime, OGDRAW, DnaSP v6.12.03, MAFFT, RAxML, and jModelTest v2.1.4, as cited in the Methods section.

## Ethics and consent

This study did not involve human participants or animals. Ethical approval and informed consent were therefore not required.

## Data Availability

NCBI GenBank: *Lepidium olgae* cp genome. Accession number PV605702. The accession numbers of comparative cp genomes used in this study are provided in Table 1.
