# Supplementary material for: The Complete Chloroplast Genome of  Lepidium olgae, a Narrow Endemic Species of the Nuratau Range, Uzbekistan
Source: F1000Res. 2026 Aug 29;15:841. Originally published 2026 Jun 1. [Version 2] doi: 10.12688/f1000research.179708.2 (PMC13768709; doi:10.12688/f1000research.179708.2)
Supplement: Peer review history

# Reviewer report: Reviewer response for version 2

**DOI:** 10.5256/f1000research.207555.r515810

**Published:** 2026-11-13

**Authors:** Abdullah Abdullah

**Affiliations:** 1 Tianjin University of Traditional Chinese Medicine, Tianjin, China

The authors address my comments and the article is now suitable for indexing.

Are the datasets clearly presented in a usable and accessible format, and the assembly and annotation available in an appropriate subject-specific repository?

Partly

Are sufficient details of the sequencing and extraction, software used, and materials provided to allow replication by others?

Partly

Are the rationale for sequencing the genome and the species significance clearly described?

Yes

Are the protocols appropriate and is the work technically sound?

Partly

Reviewer Expertise:

Plant Genomics and Evolution

I confirm that I have read this submission and believe that I have an appropriate level of expertise to confirm that it is of an acceptable scientific standard.

# Reviewer report: Reviewer response for version 2

**DOI:** 10.5256/f1000research.207555.r515811

**Published:** 2026-08-31

**Authors:** Iqbal Hussain

**Affiliations:** 1 Zhejiang University, Hangzhou, China

I have carefully reviewed the revised manuscript, **“The Complete Chloroplast Genome of** ***Lepidium olgae*** **, a Narrow Endemic Species of the Nuratau Range, Uzbekistan” (Version 2)**, and the authors’ point-by-point responses to my previous comments.

 I am satisfied with the revisions made. Authors responded to all questions and issues raised earlier by addressing the problems with duplicated plastome accessions, clarifying their sampling and nucleotide-diversity analysis, providing better information about sequencing, correcting the *ycf1* coding region and separating it from the adjacent noncoding regions, tempering the molecular markers' and tree phylogeny observations and analyses, and giving more balanced conclusion about the conservation importance and limitations of the findings.

 Thus, the revised manuscript becomes clearer, more transparent, and more scientifically sound. They have also made the correct decision to restrict their conclusions to the studied taxa and to point out the limitations of the plastome-only data.

Are the datasets clearly presented in a usable and accessible format, and the assembly and annotation available in an appropriate subject-specific repository?

Yes

Are sufficient details of the sequencing and extraction, software used, and materials provided to allow replication by others?

Partly

Are the rationale for sequencing the genome and the species significance clearly described?

Yes

Are the protocols appropriate and is the work technically sound?

Yes

Reviewer Expertise:

Gene Functional Analaysis, Genome mining, Disease resistance genetics

I confirm that I have read this submission and believe that I have an appropriate level of expertise to confirm that it is of an acceptable scientific standard.

# Reviewer report: Reviewer response for version 1

**DOI:** 10.5256/f1000research.198249.r500493

**Published:** 2026-07-23

**Authors:** Iqbal Hussain

**Affiliations:** 1 Zhejiang University, Hangzhou, China

This manuscript reports the complete chloroplast genome of *Lepidium olgae* and compares it with 16 other *Lepidium* species using plastome-based comparative analysis and phylogeny. The study provides a useful genomic resource for a poorly studied endemic Brassicaceae species. However, the current manuscript remains largely descriptive and would benefit from stronger evolutionary interpretation, more rigorous sampling justification, and improved integration of plastome biology with the known complexity of *Lepidium* evolution. Several conclusions should be moderated because chloroplast genomes represent only a single maternally inherited genetic compartment and may not fully reflect species evolutionary history, especially in a genus affected by hybridization, polyploidy, and possible reticulate evolution.

**1. Phylogenetic interpretation of Lepidium olgae requires deeper discussion**

 The authors recovered *L. olgae* as a distinct lineage based on complete chloroplast genomes (Abstract; Figure 5). However, because *Lepidium* has a complex evolutionary history involving polyploidy, hybridization, and possible reticulate evolution, plastome-based phylogeny alone may not represent the complete species history. The discussion should compare the plastome topology with previous nuclear marker studies (ITS/ETS) and clarify whether the position of *L. olgae* agrees with known Eurasian/Central Asian lineages.

**2. Taxonomic sampling is limited for genus-wide conclusions**

 The study analyzed 17 *Lepidium* species using 34 plastome accessions (Table 1), but *Lepidium* contains more than 175–260 species. Therefore, conclusions regarding overall genus evolution and taxonomy should be moderated. The authors should clearly state that the phylogenetic results represent relationships among currently available plastome datasets rather than a comprehensive genus-wide reconstruction.

 3. Duplicate accession issue in comparative analysis (Table 1)

 Table 1 includes multiple accessions for some species, for example:

- *Lepidium sativum*: NC_047178 and MK637743
- *Lepidium ferganense*: NC_077505, ON598364, and OQ644478
- *Lepidium apetalum*: NC_051540, OR941701, and PP234589

The authors should verify whether these represent independent plastomes or duplicate records of the same genome. Including identical or nearly identical plastomes may artificially increase bootstrap support in Figure 5 and influence nucleotide diversity analysis.

**4. Chloroplast genome characterization should be expanded**

 The authors report that all analyzed plastomes contain 127 unique genes (82 protein-coding genes, 37 tRNAs, and 8 rRNAs) and show high structural conservation (Results; Figure 2). However, the manuscript lacks a detailed genome feature table.

 The authors should provide:

- total genome size,
- LSC, SSC, and IR lengths,
- total GC content,
- GC content of coding genes, tRNAs, and rRNAs,
- codon usage and RSCU values,
- amino acid composition,
- SSR distribution.

These analyses are standard components of complete chloroplast genome reports and would improve the genomic characterization of *L. olgae*.

**5. ycf1 hotspot analysis requires more detailed evaluation**

 The sliding-window analysis identified the **ycf1–trnN region** as the most variable plastome region, with Pi values approximately 0.045–0.047 (Results, Figure 4). This is an important finding; however, the analysis combines a coding region ( *ycf1*) with an intergenic spacer.

 The authors should analyze separately:

1. the *ycf1* gene region,
2. the *ycf1–trnN* intergenic spacer.

Additional analyses such as Ka/Ks ratio, amino acid variation, and nucleotide diversity of *ycf1* among *Lepidium* species would provide stronger evidence for its evolutionary significance.

**6. Molecular marker potential should be validated**

 The authors suggest that variable regions such as *ycf1–trnN*, *trnQ–psbK*, *trnL–trnF*, and *rpl32–ccsA* may serve as useful molecular markers (Results; Figure 4). However, this conclusion requires comparison with commonly used Brassicaceae markers, including:

- *matK*,
- *rbcL*,
- *trnL-F*,
- ITS.

The authors should compare nucleotide diversity, variable sites, and phylogenetic resolution among these markers before recommending new barcode regions.

**7. Plastome assembly and sequencing information is insufficient**

 The Methods section states that clean reads were assembled using NOVOPlasty and annotated using Geneious Prime (Methods, lines 139–147). However, important reproducibility information is missing.

 The authors should provide:

- sequencing platform (e.g., Illumina NovaSeq/HiSeq),
- number of raw reads,
- read length,
- quality filtering software,
- trimming parameters,
- plastome coverage depth,
- assembly validation method.

Read mapping analysis showing coverage across the circular plastome would strengthen confidence in the assembly.

**8. IR boundary analysis needs quantitative comparison**

 The authors analyzed LSC/SSC/IR junctions using IRscope (Methods, lines 160–165; Figure 3). The results describe minor variation in *rps19*, *ndhF*, and *ycf1* boundaries (Results, lines 233–237).

 However, Figure 3 is mainly descriptive. The authors should provide numerical information, including:

- exact IR lengths,
- boundary coordinates,
- length differences among species,
- size of *ycf1* extension into IRa,
- *rps19* duplication length.

This would provide a stronger comparative plastome analysis.

**9. Interpretation of nucleotide diversity analysis requires clarification**

 The authors calculated nucleotide diversity using DnaSP with an 800 bp sliding window and 200 bp step size (Methods, lines 166–168). However, whole-plastome alignment may introduce uncertainty, especially in rapidly evolving intergenic regions.

 The authors should clarify:

- whether Pi analysis used all 34 accessions or one accession per species,
- how alignment gaps were treated,
- whether ambiguous regions were removed before analysis.

Feature-based analysis of individual genes and intergenic spacers would provide more reliable marker identification.

**10. Conservation significance of Lepidium olgae should be strengthened**

 Because *L. olgae* is described as a narrow endemic species from the Nuratau Range, Uzbekistan (Abstract; Figure 1), the manuscript should discuss its conservation importance more clearly. However, conclusions should remain cautious because a single plastome sequence cannot represent population-level genetic diversity.

 Future population studies using nuclear markers or genome-wide approaches should be suggested.

**11. Literature discussion should be more balanced**

 The Introduction and Discussion include several general chloroplast genome references and multiple self-citations. The manuscript would benefit from more discussion of recent *Lepidium*-specific plastome studies and previous taxonomic work. The evolutionary discussion should focus more on *Lepidium* diversification, Central Asian lineages, and plastome limitations.

 12. At present, the Results section contains both description of findings and interpretation of evolutionary significance (e.g., plastome divergence, ycf1 variation, and phylogenetic implications; Figure 4 and Figure 5). Separating Results from Discussion would improve the logical flow by allowing the Results section to focus on objective observations, while the Discussion section can provide biological interpretation, comparison with previous Lepidium studies, and evolutionary implications.A clearer structure such as:

1. Introduction
2. Materials and Methods
3. Results
  - Chloroplast genome features
  - Comparative plastome analysis
  - IR boundary variation
  - Nucleotide diversity and hotspot identification
  - Phylogenetic relationships
4. Discussion
5. Conclusions

would improve readability and make the manuscript more consistent with standard plant genomics and plastome research articles

 The manuscript presents a valuable first plastome resource for *Lepidium olgae*. The sequencing and comparative framework are appropriate; however, additional analyses, improved methodological transparency, and more cautious evolutionary interpretation are required. Addressing these issues would significantly strengthen the contribution of this study to Brassicaceae plastome genomics and phylogenetic research.

Are the datasets clearly presented in a usable and accessible format, and the assembly and annotation available in an appropriate subject-specific repository?

Yes

Are sufficient details of the sequencing and extraction, software used, and materials provided to allow replication by others?

Partly

Are the rationale for sequencing the genome and the species significance clearly described?

Yes

Are the protocols appropriate and is the work technically sound?

Yes

Reviewer Expertise:

Gene Functional Analaysis, genome mining, Haplotype analysis

I confirm that I have read this submission and believe that I have an appropriate level of expertise to confirm that it is of an acceptable scientific standard, however I have significant reservations, as outlined above.

# Response

**Published:** 2026-07-28

**Authors:** Ibrokhimjon Ergashov

**Affiliations:** Botany, Institute of Botany of the Academy of Sciences of the Republic of Uzbekistan, Tashkent, Tashkent Province, Uzbekistan

We sincerely thank the reviewer for the careful evaluation of our manuscript and for the constructive comments, which have helped improve the clarity and quality of the study. Below we provide point-by-point responses.

**Comment 1. Phylogenetic interpretation of** ***Lepidium olgae*** **requires deeper discussion**

 The authors recovered *L. olgae* as a distinct lineage based on complete chloroplast genomes. However, because *Lepidium* has a complex evolutionary history involving polyploidy, hybridization, and possible reticulate evolution, plastome-based phylogeny alone may not represent the complete species history. The discussion should compare the plastome topology with previous nuclear-marker studies and clarify whether the position of *L. olgae* agrees with known Eurasian or Central Asian lineages.

**Response.** We thank the reviewer for this important observation. We agree that a plastome-based phylogeny represents the evolutionary history of a single, predominantly maternally inherited genomic compartment and should not be interpreted as a complete species-level phylogeny.

 The interpretation of the phylogenetic results has therefore been revised to emphasize that the recovered topology represents plastome relationships among the sampled taxa. Previous phylogenetic studies based on chloroplast and nuclear ITS/ETS markers are now discussed more explicitly. However, because *L. olgae* has not been adequately represented in previous nuclear-marker datasets, its precise correspondence to a previously recognized major infrageneric lineage cannot currently be tested directly.

 The placement of *L. olgae* is now described cautiously as being biogeographically compatible with the sampled Eurasian and Central Asian representatives. We also note that hybridization, polyploidization, plastid capture, reticulate evolution, and incomplete lineage sorting may produce discordance between plastid and nuclear phylogenies. Accordingly, broader taxonomic sampling and nuclear or genome-wide data will be required to determine the species-level evolutionary position of *L. olgae* more confidently.

**Comment 2. Taxonomic sampling is limited for genus-wide conclusions**

 The study analyzed only a small proportion of the total diversity of *Lepidium* . Therefore, conclusions regarding the evolution, taxonomy, and biogeography of the entire genus should be moderated.

**Response.** We agree with the reviewer. The revised dataset contains 17 non-redundant *Lepidium* plastome accessions, representing 16 named species and one unidentified taxon, *Lepidium* sp. XJ-151. Four plastomes from *Hornungia* and *Yinshania* were used exclusively as outgroups in the phylogenetic analysis.

 Because these 17 taxa represent only a small proportion of the approximately 175–260 species currently recognized in *Lepidium* , all statements implying a comprehensive reconstruction of genus-wide evolution or biogeography have been revised. The Abstract, Results, Discussion, and concluding statements now refer specifically to the sampled taxa rather than to the genus as a whole.

 We have also added an explicit limitation stating that the available plastome sampling is taxonomically and geographically incomplete, particularly with respect to the diverse American and Australasian lineages. Consequently, the phylogenetic results are interpreted only within the context of the plastomes included in the present study.

**Comment 3. Duplicate accession issue in comparative analysis**

 Table 1 includes multiple accessions for several species, some of which may represent RefSeq and source GenBank records of the same underlying plastome. Including duplicate records may affect bootstrap support and nucleotide-diversity estimates.

**Response.** We thank the reviewer for identifying this fundamental issue. We carefully re-examined all accessions included in the original dataset and verified the relationships between NCBI RefSeq records and their corresponding source GenBank records.

 This examination confirmed that the following five accession pairs represented the same underlying plastome:

- NC_077508 and ON598368 for *Lepidium chalepense* ;
- NC_034363 and KY231152 for *L. meyenii* ;
- NC_077504 and ON598356 for *L. ruderale* ;
- NC_047178 and MK637743 for *L. sativum* ;
- NC_077505 and ON598364 for *L. ferganense* .

The redundant source GenBank records were removed, and the corresponding curated RefSeq records were retained. To prevent unequal representation of species from influencing nucleotide-diversity estimates and phylogenetic branch support, we subsequently adopted a conservative one-accession-per-taxon sampling strategy.

 The final dataset therefore contains 21 complete plastomes: 17 non-redundant *Lepidium* accessions and four outgroup accessions. Table 1 has been revised accordingly. The sequence alignment, nucleotide-diversity analysis, and maximum-likelihood phylogenetic reconstruction were repeated using the corrected dataset, and all associated figures, values, captions, and interpretations were updated.

**Comment 4. Chloroplast genome characterization should be expanded**

 The manuscript should provide a detailed genome-feature table, codon-usage and RSCU analyses, amino-acid composition, and SSR distribution.

**Response.** We thank the reviewer for this detailed recommendation. In response, we added a dedicated genome-feature table for the newly assembled plastome of *Lepidium olgae* . The table summarizes the total plastome length, LSC length, SSC length, IR length, total GC content, and regional GC contents. We also added a separate gene-content table listing the protein-coding genes, tRNA genes, and rRNA genes according to their functional categories.

 We agree that codon-usage analysis, relative synonymous codon usage, amino-acid frequency analysis, and SSR detection may provide additional information on compositional bias and potential molecular-marker development. However, the primary objective of the present Genome Note is to report the newly assembled plastome of *L. olgae* and provide a concise characterization of its genome structure, gene content, nucleotide composition, nucleotide-diversity patterns, and phylogenetic position.

 Therefore, codon-usage, RSCU, amino-acid frequency, and SSR analyses were not included in the present revision because these analyses would substantially expand the scope of the manuscript beyond its principal Genome Note objective. We have avoided claiming that these analyses were performed and acknowledge them as useful directions for a future comprehensive comparative-plastomics study.

**Comment 5. The** ***ycf1*** **hotspot requires more detailed evaluation**

 The original analysis combined the coding *ycf1* region with the adjacent *ycf1–trnN* intergenic spacer. These regions should be analyzed separately. Ka/Ks and amino-acid variation analyses may also strengthen the evolutionary interpretation.

**Response.** We thank the reviewer for this valuable methodological observation. The original whole-plastome sliding-window analysis was replaced with a feature-level nucleotide-diversity analysis using the Chloroplast Genome Analysis Suite, CGAS.

 Before the analysis, annotations were reviewed and standardized. Individual protein-coding genes, introns, tRNA genes, rRNA genes, and intergenic spacers were extracted and aligned separately. Consequently, the *ycf1* coding region and adjacent non-coding regions are now treated as distinct genomic features rather than as a combined interval.

 The revised results identify *ycf1* as one of the most variable coding regions, whereas the most variable intergenic spacers are reported separately. Because the present dataset includes only 17 sampled *Lepidium* taxa and *ycf1* may overlap the SSC/IR boundary in some plastomes, we have avoided interpreting its elevated variability as evidence of positive selection or adaptive evolution.

 Formal Ka/Ks and amino-acid substitution analyses were not included because such analyses require carefully validated orthology, codon-aware alignments, and broader taxonomic sampling. The revised manuscript therefore describes *ycf1* only as a candidate variable coding region requiring further evaluation.

**Comment 6. Molecular-marker potential should be validated**

 The proposed variable regions should be compared with commonly used markers such as *matK* , *rbcL* , *trnL-F* , and ITS before being recommended as molecular markers.

**Response.** We agree that the original wording was stronger than justified by the available evidence. The identified variable regions have not been experimentally validated as DNA barcodes or population-genetic markers.

 Accordingly, the terms “useful molecular markers” and “promising DNA barcodes” have been replaced with the more cautious expression “candidate loci for future marker development.” The revised text now states explicitly that primer design, amplification success, sequence quality, intraspecific sampling, and species-discrimination tests are required before any of these regions can be recommended as validated markers.

 We also clarify that ITS is a nuclear ribosomal marker and cannot be recovered or directly analyzed from the plastome sequences used in the present study. Previous ITS/ETS studies are therefore discussed in the phylogenetic context rather than directly reanalyzed.

 A formal comparative assessment of variable sites, parsimony-informative sites, amplification performance, and phylogenetic resolution among *matK* , *rbcL* , *trnL-F* , ITS, and the newly identified loci was considered beyond the scope of this concise Genome Note.

**Comment 7. Plastome assembly and sequencing information is insufficient**

 The manuscript should provide sequencing-platform information, number and length of raw reads, quality-filtering procedures, trimming parameters, coverage depth, assembly validation, and raw-read deposition.

**Response.** We thank the reviewer for emphasizing methodological reproducibility. The Materials and Methods section has been expanded to provide the available sequencing and assembly information.

 The revised manuscript now specifies that sequencing was performed using an Illumina NovaSeq 6000 platform with a 150-bp paired-end configuration. Information on library preparation, approximate insert size, assembly software, NOVOPlasty k-mer value, and the reference plastome used for seed selection has also been added.

 The raw paired-end sequencing reads have been deposited in the NCBI Sequence Read Archive under the following identifiers:

- BioProject: PRJNA1499946;
- BioSample: SAMN61898456;
- SRA experiment: SRX34474248;
- SRA run: SRR39796388.

The assembled and annotated plastome remains available in GenBank under accession PV605702.

 The sequencing report available to the authors did not contain all detailed preprocessing and final coverage statistics requested by the reviewer. We therefore did not invent or estimate unavailable raw-read counts, trimming parameters, or mean coverage values. Instead, the publicly deposited raw reads allow independent read mapping and assembly validation by other researchers. The Data Availability section has been revised to include the relevant GenBank and SRA identifiers.

**Comment 8. IR-boundary analysis needs quantitative comparison**

 The IR-boundary comparison should provide exact IR lengths, junction coordinates, gene-extension lengths, and numerical comparisons among species.

**Response.** We thank the reviewer for this observation. We agree that the previous IR-boundary comparison was largely qualitative and did not provide the level of numerical detail required for a rigorous comparative structural analysis.

 Because the present manuscript is intended as a concise Genome Note focused primarily on reporting the newly assembled plastome of *Lepidium olgae* , we removed the comparative LSC/SSC/IR-junction section, the associated IRscope figure, and the related qualitative interpretations from the revised manuscript.

 A detailed quantitative analysis of IR expansion and contraction—including exact junction coordinates, gene-extension lengths, pseudogene fragments, and comparisons across a broader taxonomic sample—will be more appropriately addressed in a future comparative-plastomics study.

**Comment 9. Interpretation of nucleotide-diversity analysis requires clarification**

 The authors should clarify the number of accessions included in the Pi analysis, gap treatment, removal of ambiguous regions, and whether individual genomic features were analyzed separately.

**Response.** We thank the reviewer for pointing out this ambiguity. The revised nucleotide-diversity analysis included exactly 17 non-redundant *Lepidium* plastome sequences, comprising 16 named species and one unidentified taxon, *Lepidium* sp. XJ-151.

 The four outgroup plastomes from *Hornungia* and *Yinshania* were excluded from the nucleotide-diversity analysis and were used only for phylogenetic reconstruction.

 Before analysis, plastome annotations were reviewed and standardized, including gene names, exon–intron boundaries, duplicated genes in the inverted-repeat regions, and intergenic-spacer coordinates. CGAS was then used to extract and align individual coding sequences, introns, RNA genes, and intergenic spacers separately.

 This feature-level approach replaced the original whole-plastome sliding-window analysis and reduced the risk of alignment uncertainty arising from non-homologous or structurally heterogeneous non-coding regions. The Materials and Methods, Results, figure caption, and interpretation have been revised to report the sample size and analytical approach explicitly.

**Comment 10. Conservation significance of** ***Lepidium olgae*** **should be strengthened**

 The manuscript should discuss the conservation relevance of this narrow endemic species, while recognizing that one plastome cannot represent population-level diversity.

**Response.** We agree with the reviewer. The conservation-related interpretation has been revised to emphasize both the value and the limitations of the newly generated plastome.

 The plastome sequence of *Lepidium olgae* is now described as an important reference genomic resource for a geographically restricted Central Asian endemic. However, we explicitly state that a plastome obtained from a single sampled individual cannot provide information on population-level genetic diversity, gene flow, demographic history, population structure, or adaptive variation.

 We therefore avoid making conclusions about the conservation status or genetic health of the species based solely on the present sequence. Future studies should include geographically replicated sampling from different parts of the Nuratau Range and should use nuclear markers, genome-wide SNP data, ecological information, and population-genetic approaches.

**Comment 11. Literature discussion should be more balanced**

 The Introduction and Discussion contain several general plastome references and multiple self-citations. More *Lepidium* -specific plastome, taxonomic, and phylogenetic literature should be included.

**Response.** We thank the reviewer and agree with this recommendation. The Introduction and interpretative parts of the manuscript have been revised to provide a more balanced review of the available literature.

 Non-essential self-citations were removed, and only those directly relevant to the methods or comparative context were retained. Additional independent studies on *Lepidium* taxonomy, chloroplast DNA phylogeny, nuclear ITS/ETS evidence, polyploidy, biogeography, and plastome-based analyses have been incorporated.

 The revised manuscript now places greater emphasis on the complex evolutionary history of *Lepidium* , the Eurasian and Central Asian context of *L. olgae* , and the limitations of plastome-only phylogenetic inference. Broad statements about the whole genus have also been moderated to reflect the limited taxonomic sampling of the present study.

**Comment 12. Results and Discussion should be separated**

 The reviewer recommends reorganizing the manuscript into separate Results, Discussion, and Conclusions sections.

**Response.** We thank the reviewer for this structural recommendation. We agree that separating objective observations from biological interpretation is generally beneficial in full-length comparative-genomics articles.

 However, the present article was prepared as a concise Genome Note, whose primary purpose is to report and characterize a newly sequenced plastome rather than to provide an extensive genus-wide evolutionary analysis. For this reason, we retained a compact manuscript structure rather than expanding the article into a full comparative-genomics paper with multiple additional analytical sections.

 Nevertheless, the Results section was revised to present factual observations and numerical findings more clearly before providing brief interpretation. Speculative statements were removed or moderated, broad genus-wide conclusions were restricted to the sampled taxa, and the final paragraphs were revised to summarize the principal findings, study limitations, and future research needs.

 We believe that this revised structure improves the logical flow and readability while remaining appropriate for the Genome Note article type.

 We again thank the reviewer for the thoughtful and constructive recommendations. These comments helped us improve the accuracy of the dataset, methodological transparency, cautious interpretation of plastome-based relationships, and presentation of the limitations and significance of the study.

# Reviewer report: Reviewer response for version 1

**DOI:** 10.5256/f1000research.198249.r490381

**Published:** 2026-06-13

**Authors:** Abdullah Abdullah

**Affiliations:** 1 Tianjin University of Traditional Chinese Medicine, Tianjin, China

This manuscript reports the complete chloroplast genome of Lepidium olgae which is a narrow endemic species from the Nuratau Range of Uzbekistan, and it has medicinal potential which is also documented. The work is addressing a genuine gap because Lepidium is still under sampled genomically despite its taxonomic complexity and economic importance and there was no genomic data available for this species before this study. The core rationale is sound, and the biological material is well vouchered with TASH0007 and identified by N. Beshko. However, the manuscript in its current form contains several internal inconsistencies and methodological omissions which are preventing it from meeting the reproducibility standards which is expected for a Genome Note. I am outlining these concerns below.

 The most serious problem is regarding the multiple accessions which are included in the comparative analysis Table 1. I notice that several species have been represented by more than one GenBank accession and in some cases both the NC_ RefSeq accession and another raw GenBank accession for what appears to be the same genome have been included. For example, Lepidium sativum is listed as NC_047178 and MK637743 which are likely representing the same genome under different accession numbers because NCBI assigns a RefSeq accession to an existing GenBank record. Similarly, Lepidium ferganense is given as NC_077505 alongside ON598364 and OQ644478 which means three accessions may actually correspond to no more than two distinct genomes. If the same genome is being included twice in the alignment, then it will artificially inflate branch support in the phylogeny and it will bias the Pi analysis as well. The authors must check each species carefully and they must verify which accessions represent independent genomes and which are duplicates. Only one accession per distinct genome should be retained in the alignment. This is a fundamental issue.

 The methods section is insufficiently detailed in several respects. There is no description is given of how raw reads were preprocessed before assembly and the text simply states that "Clean reads were assembled using NOVOPlasty" without mentioning quality trimming or adapter clipping or any filtering steps. Neither the sequencing platform for example NovaSeq 6000 versus HiSeq X or read length or number of raw reads generated nor the final depth of coverage for the plastome assembly is reported. The seed sequence which is used for NOVOPlasty is also not specified which is a critical detail for reproducibility because the choice of seed can influence assembly completeness. Beyond this only the assembled genome has been deposited in GenBank with accession PV605702, but the raw sequencing reads have not been submitted to the SRA under a BioProject which is a standard expectation for organellar genome publications and it is essential for reproducibility. All of these details need to be provided, and the SRA accession also need to be added in the data availability statement.

 A further methodological concern is involving the nucleotide diversity Pi analysis. The method says that DnaSP was used with a sliding window approach of 800 bp window and 200 bp step size after a whole-genome alignment with MAFFT. However, a whole-genome alignment of 30 chloroplast genome accessions from 17 species is error-prone because alignment ambiguity accumulates across the full genome particularly in highly variable intergenic regions and this will affect the reliability of the Pi estimates. A more accurate approach is to align each gene intron and intergenic spacer separately which avoids the alignment noise from non-homologous regions. I would recommend using a dedicated pipeline which is CGAS the Chloroplast Genome Analysis Suite which calculates nucleotide diversity at the level of each individually aligned annotated genomic feature and it also generates high-quality visualization figures of the Pi distribution. CGAS also integrates a more robust assembly pipeline using fastp and GetOrganelle and BWA plus Samtools for read mapping and coverage validation and it packages analysis outputs into structured datasets which is suitable for deposition on Figshare or Zenodo which would address the reproducibility gap noted above. However, for the per-feature Pi calculation in CGAS to produce reliable results the authors must first ensure that their gene annotations are correct because any annotation errors will propagate into the Pi values. Additionally, the authors must specify exactly how many of the 17 Lepidium species were included in the Pi alignment because the methods currently say "17 Lepidium species" were sampled but the Table 1 shows 30 accessions from 17 species so it is unclear whether all 30 accessions or only one per species was used for the sliding window analysis.

 A significant omission is that the manuscript does not provide any dedicated table of genome features for the newly assembled plastome of L. olgae. For a Genome Note which is reporting a new cp genome it is standard practice to present a comprehensive summary which includes the overall genome size the LSC length the SSC length the IR length the total GC content and the GC content of each region separately. It is also standard to report the GC content of protein-coding genes transfer RNAs and ribosomal RNAs individually because these values are informative for genome characterisation and they are routinely included in comparable publications. Additionally, the authors have not performed any codon usage analysis or amino acid frequency analysis which would be expected for a newly sequenced plastome to characterise its compositional bias and translational constraints. Similarly, no simple sequence repeat SSR analysis has been conducted which is a routine component of chloroplast genome characterisation and it is useful for marker development for population genetics and species identification. These three analyses which is genome features table and codon usage and SSR analysis are standard expectations for a cp genome note and their absence represent a substantial gap in the basic genomic description. A comparative table which is showing these features across all 17 Lepidium species would be even more valuable and it would strengthen the comparative framework of the manuscript considerably. All of these missing analyses including genome feature tabulation and GC content breakdown by gene category and codon usage and RSCU and amino acid frequency and SSR detection and visualisation can be performed with CGAS the Chloroplast Genome Analysis Suite which generates them in a single automated pipeline along with the nucleotide diversity analysis noted above. CGAS would also provide a comparative genome features table across multiple species which could replace the data the authors currently lack. I strongly recommend that the authors consider using CGAS to generate all of these missing standard outputs.

 A number of smaller but noticeable issues are affecting data presentation. The genome map Figure 2 caption is minimal, and it does not explain what the grayscale shading or the inner histogram values represent apart from a single sentence about GC content. The figure also lacks a legend for the gene color coding which is standard in OGDRAW outputs. The phylogenetic tree Figure 5 caption is showing a double period at the end like "Brassicaceae, including Yinshania and Hornungia, were used as outgroups.." which is a typographical error. The bootstrap values are correctly reported between 67% and 100% in this version which is an improvement. However, a gene content table which listing protein-coding genes and tRNAs and rRNAs by functional category would be a standard addition for a cp genome note. Also, the IR boundary comparison in Figure 3 is showing the boundary shifts but the discussion of these shifts remains entirely qualitative and no numerical comparison with other Lepidium species is given which limits the value of the comparative analysis.

 Several typographical errors and language issues should be corrected. The header metadata on page 1 footer says "Last updated: 02 JUN 2026" with an uppercase JUN which is inconsistent with the standard date format. In the Results section the phrase "chloroplast genome" is used throughout when "cp genome" would be sufficient after first mention following the field convention. The Introduction section contains a very long string of self-citations which is Ergashov et al. 2026a to 2026c and 2025a to 2025b which is appearing seven times in a single paragraph, and this is excessive for a Genome Note where the focus should be on the study species rather than the authors own publication record. Some of these citations also predate the study period and they do not appear directly relevant to Lepidium plastome research so I would recommend keeping only the most relevant citations and reducing the rest.

 Beyond the issue of excessive self-citations, the manuscript could benefit from a more complete review of relevant prior literature about Lepidium chloroplast genomes and phylogeny. There is a growing body of published work on cp genome sequencing and phylogenetic analysis within the genus which has appeared in various journals in recent years. The authors could cite more of these relevant studies on Lepidium plastomes to better contextualise their own findings. The current Introduction relies somewhat heavily on a few general references and on the authors own previous work and it would be improved by a broader engagement with the existing cp genome literature on Lepidium. This would help the authors to position their results more accurately within the current state of knowledge about Lepidium chloroplast genomics.

 The phylogenetic results are presented adequately but the discussion is too brief regarding the specific position of L. olgae. The tree is placing L. olgae as a distinct lineage which is said to highlight geographic isolation but the text does not explain which of the major Lepidium clades which is described by Mummenhoff et al. (2001) this correspond to and nor it is mention that whether the placement is biogeographically coherent for a Central Asian endemic. A more substantive discussion of the phylogenetic position is warranted and a supplementary tree with branch lengths would be helpful. The authors also mention "autogamous reproductive systems" as an explanation for moderate plastome divergence but they do not provide any evidence that L. olgae itself is autogamous and this claim is speculative without supporting citation or empirical data. The Discussion section is also over-reliant on self-citations which is giving an impression of self-promotion rather than a balanced engagement with the wider literature. Furthermore, the authors are including only 17 species out of more than 175 to 260 species in Lepidium which represents a relatively small fraction of the genus. The sampling is also geographically limited with no representatives from the Australasian and American clades which are known to harbour substantial diversity. Given this limited taxonomic and geographic coverage broad conclusions about the phylogeny and biogeography of the entire genus should not be drawn and the claims in the Discussion should be restricted to the sampled taxa.

 Taken together this manuscript is describing a useful new genomic resource for a species which genuinely needed one and the core sequencing and analytical work is on the right track. However the duplicate accessions problem in Table 1 which is potentially introducing identical genomes into the comparative analysis and the absence of critical methodological details like read preprocessing and coverage depth and SRA deposition and the inadequate sliding window approach which misses gene-level Pi resolution and the missing genome features table with GC content and LSC SSC and IR sizes and GC content by gene category and the absence of codon usage amino acid frequency and SSR analyses which are standard for a Genome Note and the excessive self-citations and the insufficiently detailed figures and the number of typographical errors all need to be addressed before the manuscript can be accepted. As noted above many of these gaps including Pi analysis at the gene level and genome feature tabulation and GC content breakdown and codon usage and SSR detection can all be resolved using CGAS the Chloroplast Genome Analysis Suite which would also help standardise the data outputs and improve reproducibility. I am recommending major revisions for the manuscript.

Are the datasets clearly presented in a usable and accessible format, and the assembly and annotation available in an appropriate subject-specific repository?

Partly

Are sufficient details of the sequencing and extraction, software used, and materials provided to allow replication by others?

Partly

Are the rationale for sequencing the genome and the species significance clearly described?

Yes

Are the protocols appropriate and is the work technically sound?

Partly

Reviewer Expertise:

Plant Genomics and Evolution

I confirm that I have read this submission and believe that I have an appropriate level of expertise to confirm that it is of an acceptable scientific standard, however I have significant reservations, as outlined above.

# Response

**Published:** 2026-07-28

**Authors:** Ibrokhimjon Ergashov

**Affiliations:** Botany, Institute of Botany of the Academy of Sciences of the Republic of Uzbekistan, Tashkent, Tashkent Province, Uzbekistan

We sincerely thank the reviewer for the careful evaluation of our manuscript and for the constructive comments, which have helped improve the clarity and quality of the study. Below we provide point-by-point responses.

**Reviewer #1**

**Comment 1.** The most serious problem is regarding the multiple accessions which are included in the comparative analysis Table 1. I notice that several species have been represented by more than one GenBank accession and in some cases both the NC_ RefSeq accession and another raw GenBank accession for what appears to be the same genome have been included. For example, Lepidium sativum is listed as NC_047178 and MK637743 which are likely representing the same genome under different accession numbers because NCBI assigns a RefSeq accession to an existing GenBank record. Similarly, Lepidium ferganense is given as NC_077505 alongside ON598364 and OQ644478 which means three accessions may actually correspond to no more than two distinct genomes. If the same genome is being included twice in the alignment, then it will artificially inflate branch support in the phylogeny and it will bias the Pi analysis as well. The authors must check each species carefully and they must verify which accessions represent independent genomes and which are duplicates. Only one accession per distinct genome should be retained in the alignment. This is a fundamental issue.

**Response.** We thank the reviewer for identifying this important issue. We carefully re-examined all 34 accession records included in the original Table 1 and checked the relationships between the NCBI RefSeq records and their corresponding source GenBank records.

 This examination confirmed that five RefSeq/GenBank accession pairs represented the same underlying plastome: NC_077508/ON598368 for *Lepidium chalepense* , NC_034363/KY231152 for *L. meyenii* , NC_077504/ON598356 for *L. ruderale* , NC_047178/MK637743 for *L. sativum* , and NC_077505/ON598364 for *L. ferganense* . The redundant source GenBank records were therefore removed, and the corresponding curated RefSeq records were retained.

 Several species were also represented by multiple additional accessions that could correspond to independently sequenced individuals. To prevent unequal representation of particular species from affecting the nucleotide-diversity estimates and phylogenetic branch support, we adopted a conservative one-accession-per-taxon sampling strategy. Where an NCBI RefSeq record was available, the curated RefSeq accession was preferentially retained. For taxa without an available RefSeq record, one complete GenBank plastome was retained.

 Following this revision, the final dataset contains 21 complete plastome sequences, comprising 17 *Lepidium* taxa—16 named species and one unidentified accession, *Lepidium* sp. XJ-151—and four outgroup taxa. Table 1 has been revised to list only the accessions included in the corrected analyses.

 Using the revised, non-redundant dataset, we repeated the complete sequence alignment, sliding-window nucleotide-diversity (Pi) analysis, and maximum-likelihood phylogenetic reconstruction. The Pi values, phylogenetic topology, bootstrap-support values, figures, tables, and all associated numerical results were regenerated. The Materials and Methods, Results, Discussion, Table 1, and the captions and descriptions of the nucleotide-diversity and phylogenetic figures have been revised accordingly.

**Comment 2.** The methods section is insufficiently detailed in several respects. There is no description is given of how raw reads were preprocessed before assembly and the text simply states that "Clean reads were assembled using NOVOPlasty" without mentioning quality trimming or adapter clipping or any filtering steps. Neither the sequencing platform for example NovaSeq 6000 versus HiSeq X or read length or number of raw reads generated nor the final depth of coverage for the plastome assembly is reported. The seed sequence which is used for NOVOPlasty is also not specified which is a critical detail for reproducibility because the choice of seed can influence assembly completeness. Beyond this only the assembled genome has been deposited in GenBank with accession PV605702, but the raw sequencing reads have not been submitted to the SRA under a BioProject which is a standard expectation for organellar genome publications and it is essential for reproducibility. All of these details need to be provided, and the SRA accession also need to be added in the data availability.

**Response.** We thank the reviewer for highlighting the insufficient methodological detail and the importance of making the raw sequencing data publicly available. We have substantially revised the Materials and Methods section to provide detailed information.

**Comment 3.** A further methodological concern is involving the nucleotide diversity Pi analysis. The method says that DnaSP was used with a sliding window approach of 800 bp window and 200 bp step size after a whole-genome alignment with MAFFT. However, a whole-genome alignment of 30 chloroplast genome accessions from 17 species is error-prone because alignment ambiguity accumulates across the full genome particularly in highly variable intergenic regions and this will affect the reliability of the Pi estimates. A more accurate approach is to align each gene intron and intergenic spacer separately which avoids the alignment noise from non-homologous regions. I would recommend using a dedicated pipeline which is CGAS the Chloroplast Genome Analysis Suite which calculates nucleotide diversity at the level of each individually aligned annotated genomic feature and it also generates high-quality visualization figures of the Pi distribution. CGAS also integrates a more robust assembly pipeline using fastp and GetOrganelle and BWA plus Samtools for read mapping and coverage validation and it packages analysis outputs into structured datasets which is suitable for deposition on Figshare or Zenodo which would address the reproducibility gap noted above. However, for the per-feature Pi calculation in CGAS to produce reliable results the authors must first ensure that their gene annotations are correct because any annotation errors will propagate into the Pi values.

**Response.** We thank the reviewer for this important methodological recommendation. We agree that nucleotide-diversity estimates based on a single whole-plastome alignment may be affected by alignment uncertainty, particularly in highly variable and structurally heterogeneous non-coding regions.

 Before repeating the nucleotide-diversity analysis, we carefully reviewed and standardized the annotations of all plastomes included in the comparative dataset. Gene names, exon–intron boundaries, duplicated genes located in the inverted-repeat regions, and the coordinates of intergenic spacers were checked to minimize errors during feature extraction. Duplicate accessions identified during revision of Table 1 were also removed before the analysis.

 We then repeated the nucleotide-diversity analysis using the Chloroplast Genome Analysis Suite, CGAS, following the feature-level approach described by Abdullah et al. (2026).

**Comment 4.** Additionally, the authors must specify exactly how many of the 17 Lepidium species were included in the Pi alignment because the methods currently say "17 Lepidium species" were sampled but the Table 1 shows 30 accessions from 17 species so it is unclear whether all 30 accessions or only one per species was used for the sliding window analysis.

**Response.** We thank the reviewer for pointing out this ambiguity. In the original analysis, multiple accessions from several *Lepidium* species were included, which resulted in unequal representation among species. Following the reviewer’s recommendation, we revised the dataset and retained only one chloroplast genome accession for each *Lepidium* taxon.

 The revised nucleotide-diversity analysis therefore included exactly 17 *Lepidium* plastome sequences, representing 16 named species and one unidentified taxon, *Lepidium* sp. XJ-151. The four outgroup plastomes were not included in the sliding-window nucleotide-diversity analysis and were used only for phylogenetic reconstruction.

 The complete plastome alignment and sliding-window Pi analysis were repeated using these 17 non-redundant *Lepidium* accessions. Table 1, the Materials and Methods section, the nucleotide-diversity figure, and all associated values and descriptions in the Results and Discussion sections have been revised accordingly.

**Comment 5.** A significant omission is that the manuscript does not provide any dedicated table of genome features for the newly assembled plastome of *L. olgae* . For a Genome Note which is reporting a new cp genome it is standard practice to present a comprehensive summary which includes the overall genome size the LSC length the SSC length the IR length the total GC content and the GC content of each region separately. It is also standard to report the GC content of protein-coding genes transfer RNAs and ribosomal RNAs individually because these values are informative for genome characterisation and they are routinely included in comparable publications.

**Response.** We thank the reviewer for identifying this important omission. In response, we have added a new table summarizing the principal structural and compositional features of the newly assembled chloroplast genome of *Lepidium olgae* .

**Comment 6.** Additionally, the authors have not performed any codon usage analysis or amino acid frequency analysis which would be expected for a newly sequenced plastome to characterise its compositional bias and translational constraints. Similarly, no simple sequence repeat SSR analysis has been conducted which is a routine component of chloroplast genome characterisation and it is useful for marker development for population genetics and species identification. These three analyses which is genome features table and codon usage and SSR analysis are standard expectations for a cp genome note and their absence represent a substantial gap in the basic genomic description. A comparative table which is showing these features across all 17 Lepidium species would be even more valuable and it would strengthen the comparative framework of the manuscript considerably. All of these missing analyses including genome feature tabulation and GC content breakdown by gene category and codon usage and RSCU and amino acid frequency and SSR detection and visualisation can be performed with CGAS the Chloroplast Genome Analysis Suite which generates them in a single automated pipeline along with the nucleotide diversity analysis noted above. CGAS would also provide a comparative genome features table across multiple species which could replace the data the authors currently lack. I strongly recommend that the authors consider using CGAS to generate all of these missing standard outputs.

**Response.** We thank the reviewer for this constructive and detailed recommendation. We agree that codon-usage analysis, relative synonymous codon usage (RSCU), amino-acid frequency analysis, and simple sequence repeat (SSR) detection can provide additional information on plastome compositional bias, potential translational constraints, and candidate molecular markers for population-genetic and species-identification studies.

 We would also like to clarify that the Chloroplast Genome Analysis Suite (CGAS) was used in the revised manuscript to perform the sliding-window nucleotide-diversity (Pi) analysis. This analysis was repeated using the corrected dataset of 17 non-redundant *Lepidium* plastomes, with one accession retained per taxon. The relevant methods, Pi values, graphical outputs, and interpretations have been updated accordingly.

 However, the primary objective of the present Genome Note was to report the newly assembled plastome of *L. olgae* and provide a concise characterization of its genome structure, gene content, nucleotide composition, nucleotide-diversity patterns, and phylogenetic position. Therefore, codon-usage and RSCU analyses, amino-acid frequency analysis, and SSR detection and visualization were not included in the present revision, as these analyses would substantially expand the scope of this Genome Note beyond its principal objective.

**Comment 7.** A number of smaller but noticeable issues are affecting data presentation. The genome map Figure 2 caption is minimal, and it does not explain what the grayscale shading or the inner histogram values represent apart from a single sentence about GC content. The figure also lacks a legend for the gene color coding which is standard in OGDRAW outputs.

**Response.** We thank the reviewer for pointing out these presentation issues. We have revised the caption of Figure 2 to provide a more complete explanation of the chloroplast genome map. The revised caption now describes the transcriptional orientation of the genes, the functional classification represented by the different gene colors, the local GC-content histogram, and the positions of the LSC, SSC, IRa, and IRb regions.

**Comment 8.** The phylogenetic tree Figure 5 caption is showing a double period at the end like "Brassicaceae, including Yinshania and Hornungia, were used as outgroups.." which is a typographical error. The bootstrap values are correctly reported between 67% and 100% in this version which is an improvement.

**Response.** We thank the reviewer for identifying this typographical error and for noting the improvement in the reported bootstrap-support values. The duplicated period at the end of the Figure 5 caption has been removed.

 In addition, to avoid unequal representation of species and the possible inflation of branch-support values, we revised the phylogenetic dataset by retaining only one chloroplast genome accession for each *Lepidium* taxon. The sequence alignment and phylogenetic reconstruction were repeated using the corrected non-redundant dataset. Figure 5, the bootstrap-support values, the figure caption, and the corresponding descriptions in the Results and Discussion sections have been updated accordingly.

**Comment 9.** However, a gene content table which listing protein-coding genes and tRNAs and rRNAs by functional category would be a standard addition for a cp genome note.

**Response.** We thank the reviewer for this valuable suggestion. In response, we have added a dedicated gene-content table for the newly assembled chloroplast genome of *Lepidium olgae* .

**Comment 10.** Also, the IR boundary comparison in Figure 3 is showing the boundary shifts but the discussion of these shifts remains entirely qualitative and no numerical comparison with other Lepidium species is given which limits the value of the comparative analysis.

**Response.** We thank the reviewer for this observation. We agree that the previous IR-boundary comparison was insufficiently quantitative. Because the present manuscript is intended as a concise Genome Note rather than a comprehensive study of plastome structural evolution, we removed the “Comparative analysis of the LSC, SSC, and IR junctions” section, the corresponding figure, and all associated text from the Results and Discussion. A detailed numerical analysis of IR expansion and contraction across *Lepidium* species will be addressed in a future comparative study

**Comment 11.** Several typographical errors and language issues should be corrected. The header metadata on page 1 footer says "Last updated: 02 JUN 2026" with an uppercase JUN which is inconsistent with the standard date format. In the Results section the phrase "chloroplast genome" is used throughout when "cp genome" would be sufficient after first mention following the field convention. The Introduction section contains a very long string of self-citations which is Ergashov et al. 2026a to 2026c and 2025a to 2025b which is appearing seven times in a single paragraph, and this is excessive for a Genome Note where the focus should be on the study species rather than the authors own publication record. Some of these citations also predate the study period and they do not appear directly relevant to Lepidium plastome research so I would recommend keeping only the most relevant citations and reducing the rest.

**Response.** We thank the reviewer for identifying these typographical, stylistic, and citation-related issues. We have carefully revised the manuscript to improve consistency, clarity, and conciseness.

 Regarding the date shown in the page metadata (“02 JUN 2026”), we would like to clarify that this information is generated automatically by the journal’s submission or publication platform and is not part of the manuscript text prepared by the authors. Therefore, its formatting is outside the authors’ direct control and may be standardized by the journal during the production stage.

 We have also revised the terminology throughout the manuscript. The term “chloroplast genome” is now written in full at its first occurrence, after which “cp genome” or “plastome” is used consistently, where appropriate, to avoid unnecessary repetition.

 In addition, we agree that the number of self-citations in the Introduction was excessive for a concise Genome Note. We therefore reassessed all citations to Ergashov et al. and removed those that were not directly relevant to *Lepidium* , cp-genome characterization, or the methodological context of the present study. Only the most relevant self-citations have been retained, and independent references have been added where appropriate. The revised Introduction now focuses more directly on the study species, the significance of the newly assembled cp genome, and the objectives of the present work.

**Comment 12.** Beyond the issue of excessive self-citations, the manuscript could benefit from a more complete review of relevant prior literature about Lepidium chloroplast genomes and phylogeny. There is a growing body of published work on cp genome sequencing and phylogenetic analysis within the genus which has appeared in various journals in recent years. The authors could cite more of these relevant studies on Lepidium plastomes to better contextualise their own findings. The current Introduction relies somewhat heavily on a few general references and on the authors own previous work and it would be improved by a broader engagement with the existing cp genome literature on Lepidium. This would help the authors to position their results more accurately within the current state of knowledge about Lepidium chloroplast.

**Response.** We thank the reviewer for this constructive suggestion. We agree that the previous version of the Introduction did not sufficiently reflect the growing body of literature on chloroplast genome sequencing, comparative plastomics, and phylogenetic relationships within *Lepidium* .

 Accordingly, we have substantially revised the Introduction and expanded the literature review by incorporating additional recent and independent studies on *Lepidium* plastomes and phylogeny. At the same time, we reduced the number of self-citations and retained only those directly relevant to the objectives and methods of the present study.

 The revised Introduction now provides a broader overview of previous chloroplast genome research in *Lepidium* , including genome structure, sequence variation, comparative plastome analyses, and plastome-based phylogenetic reconstruction. We have also clarified how the newly assembled cp genome of *L. olgae* contributes to the existing genomic resources for the genus and how the present study extends current knowledge of *Lepidium* plastome diversity and phylogenetic relationships. Relevant parts of the Discussion were also revised to compare our findings more directly with previously published studies.

**Comment 13.** The phylogenetic results are presented adequately but the discussion is too brief regarding the specific position of L. olgae. The tree is placing L. olgae as a distinct lineage which is said to highlight geographic isolation but the text does not explain which of the major Lepidium clades which is described by Mummenhoff et al. (2001) this correspond to and nor it is mention that whether the placement is biogeographically coherent for a Central Asian endemic. A more substantive discussion of the phylogenetic position is warranted and a supplementary tree with branch lengths would be helpful.

**Response.** We recalculated the phylogenetic tree using one plastome accession per species, removing additional accessions from species that had previously been represented by multiple samples. The complete plastome alignment, maximum-likelihood analysis, bootstrap-support values, Figure 5, and the corresponding descriptions in the Results and Discussion sections were updated accordingly.

**Comment 14.** The authors also mention "autogamous reproductive systems" as an explanation for moderate plastome divergence but they do not provide any evidence that L. olgae itself is autogamous and this claim is speculative without supporting citation or empirical data.

**Response.** We thank the reviewer for identifying this unsupported interpretation. We agree that there is currently no direct evidence demonstrating that *Lepidium olgae* has an autogamous reproductive system. Therefore, the statement linking its plastome divergence to autogamy was speculative and has been removed from the revised manuscript.

 The Discussion has been revised to describe the observed plastome divergence more cautiously, without attributing it to a particular reproductive system in the absence of species-specific empirical data or supporting literature.

**Comment 14.** The Discussion section is also over-reliant on self-citations which is giving an impression of self-promotion rather than a balanced engagement with the wider literature.

**Response.** We thank the reviewer for this important observation. We have therefore reviewed all self-citations and removed those that were not directly necessary to support the interpretation of the present results.

**Comment 15.** Furthermore, the authors are including only 17 species out of more than 175 to 260 species in Lepidium which represents a relatively small fraction of the genus. The sampling is also geographically limited with no representatives from the Australasian and American clades which are known to harbour substantial diversity. Given this limited taxonomic and geographic coverage broad conclusions about the phylogeny and biogeography of the entire genus should not be drawn and the claims in the Discussion should be restricted to the sampled taxa.

**Response.** We thank the reviewer for highlighting the limitations of the taxonomic and geographic sampling. We agree that the 17 sampled *Lepidium* taxa represent only a small proportion of the total diversity of the genus and are insufficient for drawing broad conclusions about the phylogeny or biogeographic history of *Lepidium* as a whole.

 The present study was designed as a Genome Note focused primarily on reporting and characterizing the complete cp genome of the narrowly distributed Central Asian endemic *Lepidium olgae* . The comparative dataset was assembled from complete *Lepidium* plastomes that were publicly available in the NCBI database at the time of the analysis and was not intended to provide comprehensive representation of all major geographic lineages within the genus
